# Towards selective pharmacological modulation of protein kinase C--opportunities for the development of novel antineoplastic agents.

**DOI:** 10.1038/bjc.1992.209

**Published:** 1992-07

**Authors:** A. Gescher


*Br. J. Cancer* (1992), **66**, 10–19 © Macmillan Press Ltd., 1992

GUEST EDITORIAL

### Towards selective pharmacological modulation of protein kinase C – opportunities for the development of novel antineoplastic agents

A. Gescher

*Cancer Research Campaign Experimental Chemotherapy Group, Pharmaceutical Sciences Institute, Aston University, Birmingham B47ET, UK.*

The biochemistry of the cell membrane and cellular signalling systems have recently come to the fore as potential targets for the discovery and development of new anticancer drugs (Tritton & Hickman, 1990; Powis, 1991). As part of this new focus natural products with potentially exploitable biological properties receive increasing attention. The marine animal product bryostatin 1 is a prototype of such agents. The most prominent biochemical feature of this investigational drug is its ability to activate the enzyme protein kinase C (PKC), and this is probably intrinsically linked to its antineoplastic activity. Bryostatin 1 is currently under phase 1 clinical evaluation in the UK, and there is considerable uncertainty as to the potential therapeutic and toxicological implications of PKC modulation. It is especially unclear how pharmacological alteration of the properties of such a crucial and ubiquitous enzyme could be selective, because normal cells, as well as their diseased counterparts, would be susceptible to disruption (Tritton & Hickman, 1990). In view of this uncertainty and of the prospect of more agents of similar structural and biological complexity entering the oncologist's chemotherapeutic armamentarium it seems appropriate to summarise the scientific basis upon which the potential use of PKC modulators such as bryostatin 1 in cancer treatment is founded. This is the aim of the commentary. In particular recently discovered characteristics of PKC activity and regulation are highlighted as far as they might eventually lead to selective intervention by drugs.

### PKC activity

The cellular effects of many growth factors and hormones are brought about by receptor-mediated hydrolysis of the membrane phospholipid phosphatidylinositol 4,5-bisphosphate. This hydrolysis results in the production of inositol 1,4,5-trisphosphate and diacylglycerol. Both molecules function as intracellular second messengers. Inositol 1,4,5-trisphosphate releases $Ca^{2+}$ from internal stores at the endoplasmic reticulum, and diacylglycerol activates PKC. PKC is an enzyme family with serine/threonine kinase function which regulates many cellular processes, including cell proliferation and differentiation (Nishizuka, 1989). PKC activity is dependent on the presence of phospholipids, and most PKC isozymes require $Ca^{2+}$ for optimal activity. Most activated PKC is found at the cell membrane, but a fraction has been located associated with the nucleus (Shimizu & Shimizu, 1989). Nuclear PKC is possibly of high functional importance as stimulation of PKC elevates, directly or indirectly, the activity of nuclear proto-oncogenes such as c-*fos*, and c-*jun*, sometimes referred to as 'immediate early genes'.

The signal transduction pathway which operates *via* PKC than other types of cancer cells (Hirai *et al.*, 1989), but

Received 14 February 1992; and in revised form 19 March 1992.

appears to be essentially ubiquitous. But the extraordinary diversity of responses to PKC activation could be explained by the multiple gene products that constitute the PKC family (α,β,γ,ε,δ,ζ,η) and their tissue-selective distribution (Nishizuka, 1988; Parker *et al.*, 1989; Stabel & Parker, 1991). Whilst it has not yet been possible to ascribe specific cellular responses to the activation of individual PKC isotypes, investigation of their properties has provided evidence for functional heterogeneity: PKC isozymes differ from each other in substrate specificity (Schaap & Parker, 1990; Marais & Parker, 1989) and in their dependency on $Ca^{2+}$ (Schaap *et al.*, 1989; Akita *et al.*, 1990) or on diacyglycerol/phospholipid (Ono *et al.*, 1989) for maximal activation.

Is there then a difference between tumours and normal tissues in levels or isozyme content of PKC, which could be therapeutically exploited? The relevant literature is not easy to interpret, which applies also to many other aspects related to PKC. PKC expression has been studied in leukaemias, melanomas, and breast, colon and lung cancers. Subtle but significant differences have been observed. Of the three major PKC isozymes α, β and γ, which were found in human leukaemia cells the former two were present in all types of leukaemic cells (Komada *et al.*, 1991). Acute myelocytic leukaemia cells expressed both types at equal levels, whereas in lymphoid cells expression of PKC-β was more abundant than that of PKC-α. PKC-γ was expressed in lymphoid leukaemia, but only sporadically in acute myeloid leukaemia cells. A comparison of murine non-transformed melanocytes and transformed melanoma cells is particularly instructive, as subcellular distribution and overall levels of PKC differed depending on cell type and proliferation state (Brooks *et al.*, 1991). In quiescent melanocytes most PKC activity was in the cytosol, whereas in the melanoma cells the majority of PKC activity was located in the membrane fraction. Total PKC levels were higher in quiescent melanocytes than in melanoma cells, which in turn contained more enzyme than proliferating melanocytes. These results indicate that overall PKC levels are related to proliferative capacity and that cytosolic enzyme might play an anti-proliferative role. Normal human melanocytes did not express PKC-α, -β, or -γ, whereas the α isozyme was expressed in primary and metastatic melanoma (Becker *et al.*, 1990). Incongruous with these results Yamanishi *et al.* (1991) did find PKC-α, -β and -ε in primary melanocytes, and they reported that PKC isotype gene expression changed during the progression of melanocytes to metastatic melanoma. PKC levels in surgical specimens of human breast tumours were almost 3-fold the enzyme activities in normal breast tissue obtained from the same patients (O'Brian *et al.*, 1989). In contrast, in human colonic adenoma or carcinoma samples PKC activity was significantly reduced as compared to adjacent mucosa tissue and mucosa in control subjects (Kopp *et al.*, 1991). PKC activity in normal colonic mucosa from patients with colorectal cancer was only a third to one half of levels in patients without cancer (Sakanoue *et al.*, 1991). Human lung carcinoma cells appear to possess higher activities of PKC-α

than other types of cancer cells (Hirai *et al.*, 1989), but PKC-α is also the most abundant isoform of PKC in normal tiusues and cells (Nishizuka, 1989). Viewed together the data on PKC levels and expression suggest that differences in PKC activity and isotype composition are dependent on origin of tissue and type of malignancy, and that generalisations as to which PKC-isozymes play a role in specific neoplasias cannot be made yet.

### PKC activators and growth arrest

In many cells activation of PKC triggers mitogenesis. However in others, mainly malignant cells of human origin, it causes the reverse, cytostatis or cytotoxicity. This observation is germane to the argument that PKC activation might be of therapeutic benefit. Therefore in the following known endogenous and exogenous PKC activators and cell types in which they cause growth arrest are briefly reviewed. Diacylglycerols are the major cellular PKC-activating ligands, certain fatty acids such as arachidonate also have some activatory ability. Upon binding to PKC diacylglycerols increase the affinity of the enzyme for $Ca^{2+}$. Research on PKC gathered momentum when the specific cellular receptor *via* which tumour promoting phorbol esters exerts its biological effects was shown to be PKC (Nishizuka, 1988). Phorbol esters, of which 12-*O*-tetradecanoylphorbol-13-acetate (TPA) (Figure 1) is the most potent, are strong activators of PKC. But they are much more resistant to metabolism than diacylglycerols, and it is the protracted stimulation of PKC by phorbol esters which is thought to trigger their effects, including tumour promotion. A fateful but intrinsic consequence of the sustained binding of biochemically stable ligands such as phorbol esters to the enzyme is its eventual demise in that they cause enzyme downregulation (Rodriguez-Pena & Rozengurt, 1984). Binding to and downregulation of PKC are at the heart of the specificity with which PKC activators exert their effects and both undoubtedly determine differences in efficacy between agents. There are several controversial issues concerning the nature of the binding of activators to PKC and the mechanism proper of activation, and they have recently been cogently reviewed (Blumberg, 1991). Marked differences in binding affinity of TPA for its receptor sites between the major PKC isozymes (α, β and γ) *in vitro* do not exist. But different phorbol esters do activate PKC isozymes somewhat disparately, consistent with the rigid structural control which governs their biological properties (Hecker, 1985; Brooks *et al.*, 1989). Six phorbol esters were recently investigated for their proficiency to activate PKC-α, -β, -γ, -δ, or -ε (Ryves *et al.*, 1991). Four agents were more or less indiscriminate in their effects on the isozymes, but two showed interesting specificity. Sapintoxin A (Figure 1) activated all isozymes except PKC-δ, whereas 12-deoxyphorbol-13-*O*-phenylacetate-20-acetate (DOPPA) (Figure 1) activated almost exclusively PKC-$\beta_1$. Also arachidonate activated PKC-α, -β and -γ differentially, depending on specific cofactors (Sekiguchi *et al.*, 1989).

Examples of cells in which phorbol esters cause cytostasis or cytotoxicity are MCF-7 breast, A431 epidermal and A549 lung carinoma cells (Gescher, 1985). Phorbol esters are also growth inhibitors of several transformed melanoma lines in culture, whereas normal melanocytes need the continual presence of phorbol esters in order to grow (Becker *et al.*, 1990; Brooks *et al.*, 1990), a finding indicative of a proreplicative role of PKC downregulation in this cell type (vide supra). How phorbol esters arrest the growth of cells is unclear, but cytostasis seems to be unquivocally linked to PKC activation. Growth arrest caused by TPA in A549 cells was dependent on the presence of foetal calf serum (Bradshaw *et al.*, 1991), which suggests the involvement of positive and/or negative growth factors. In actively dividing human B-lymphoma cells efficient interruption of the cell cycle in two places was a consequence of PKC activation (Beckwith *et al.*, 1990). In some cell lines, prominent among them the

**Figure 1** Structures of PKC activators.

HL-60 promyelocytic leukaemia, the phorbol ester-induced loss of proliferative potential is the result of induction of differentiation (Vandenbark & Niedel, 1984). TPA-induced differentation is often terminal, but in U-973 leukaemia cells it is not, as after removal of phorbol esters cells undergo retrodifferentiation (Hass *et al.*, 1991). The search for the discovery of nuclear events which trigger differentiation is on, and the TPA-induced 'immediate early' and transient transcription of a HL-60 cell gene has been reported (Shimizu *et al.*, 1991). It encodes a protein related to products of the *jun* gene family.

One inevitable conclusion of the bewildering literature on the nature and degree of response to PKC activation is that phorbol ester effects are cell-type dependent (Weinstein, 1988). PKC isozyme heterogeneity (vide supra) could account for this cell type specificity. Extrapolated to cells in which PKC activation causes changes in growth this idea would mean that certain PKC isozymes are responsible for mitogenesis, others for growth arrest. If this was so an insight into the specific roles and differential activation of PKC isozymes might provide the key to unlock the understanding of differential cellular responses to phorbol esters. Several papers, especially some on the role of oncogenes in PKC isozyme expression, hint at the contours of this key. Of the three PKC isozymes α, β and γ the β- form was by far the most abundant in the cytosol of naive HL-60 cells, whereas it was dramatically reduced in cells which had been rendered resistant towards TPA-induced differentiation by continued exposure to TPA (Nishikawa *et al.*, 1990). TPA also activated one of the two major PKC isoforms in HL-60 cells, probably PKC-β, more effectively than the other, PKC-α (Beh *et al.*, 1989). These results are consistent with the hypothesis that PKC-β is particularly important for induction of differentiation. Accordingly expression of this isozyme, but not of PKC-α, correlated with the responsiveness of KG-1 myeloid leukaemia cells to the differentiating effects of TPA (Hooper *et al.*, 1989), and 1,25-dihydroxyvitamin $D_3$-induced differentiation of HL-60 cells resulted in an increase of predominantly PKC-β transcripts (Obeid *et al.*, 1990). Rat embryo fibroblasts and liver epithelial cell lines which were transformed by an activated human c-Ha-*ras* oncogene displayed a several-fold increase in PKC-α and a concomitant decrease in PKC-ε, at both the protein and mRNA levels (Borner *et al.*, 1990). The authors tendered the intriguing hypothesis that both, upregulation of PKC-α and downregulation of PKC-ε, might be necessary for an activated *ras* gene to accomplish its transforming function, perhaps because PKC-α and PKC-ε play reciprocal roles in cell transformation, PKC-α mediating mitogenic events, whereas PKC-ε having the opposite effect. These rat cells are not the only system in which transformation by *ras* oncogenes causes PKC modulation. Ha-*ras* or Ki-*ras*-transformed NIH 3T3 fibroblasts possess constitutively elevated diacylglycerol levels with consequent partial activation and downregulation of PKC (Wolfman & Makara, 1987), although the isozymes involved have not been identified. The corollary of these findings for therapy might ultimately be that treatment of *ras*-transformed malignancies should aim at raising PKC-ε and/or inhibiting PKC-α activity. In view of the frequency of mutated *ras* in human malignancies such an approach might lead to broad-spectrum anticancer agents.

There is other evidence which supports the notion that specific PKC activators might be of therapeutic value. Phorbol ester-induced progression of initiated cells to papillomas and carcinomas was prevented by exposing cells to weak promoting regimes using TPA prior to treatment to achieve promotion proper (Reddy & Fialkow, 1990). This result would suggest that modulation of specific PKC isozymes with a high affinity activator might prevent the action of tumour promoters. Overexpression of PKC-$\beta_1$ in HT29 colon cancer cells caused growth inhibition *in vitro* (Choi *et al.*, 1990). These cells displayed decreased tumourigenicity when grown in nude mice and, unlike control transformants which lacked the PKC cDNA insert, were induced to differentiate by TPA (Choi & Weinstein, 1991). These findings led to the suggestion that in some tumours PKC might act as a growth suppressor gene. It hs to be pointed out though that in many other cell lines overexpression of PKC provokes the enhancement rather than retardation of growth rate (Krauss *et al.*, 1989; Housey *et al.*, 1988; Persons *et al.*, 1988), which highlights the complex cell- and isozyme-specificity of events catalysed by PKC.

Diacylglycerols have not shown the strong growth-inhibitory properties in the cell lines in which phorbol esters are cytostatic, perhaps due to their swift metabolic removal from the site of action. Dioctanoylglycerol (Figure 1) was not an efficacious inducer of HL-60 cell differentation (Morin *et al.*, 1987), nor did it cause cytostasis in MCF-7 (Valette *et al.*, 1987) or A549 cells (Laughton *et al.*, 1989*a*) unless relatively high cytotoxic concentrations were applied. Synthesis of analogues of diacyglycerols, in which the diester moiety is part of a ring structure to resemble ring C of the TPA molecule, or with an alkylated glycerol backbone, did not yield potent growth-inhibitory agents or compounds with high affinity for PKC (Molleyres & Rando, 1988; Laughton *et al.*, 1989*b*).

In the above examples PKC activation is linked with cytostasis more or less directly. Alternatively, PKC activators could conceivably be beneficial in cancer treatment *via* indirect mechanisms. PKC-mediated alteration of cellular sensitivity to cytotoxic drugs was shown in human ovarian carcinoma (Isonishi *et al.*, 1990) and cervical carcinoma (HeLa) cells (Basu *et al.*, 1990). Both were rendered more responsive to the antiproliferative effect of cis-diamminedichloroplatinum(II) (cis-DDP) by phorbol esters. Analogues of the non-phorbol ester promoter lyngbyatoxin A had a similar effect (Basu *et al.*, 1991) and the ability of these agents to sensitise cells to cis-DDP was correlated with their ability to activate PKC. These findings invoke the possibility to overcome anticancer drug resistance in certain cells with PKC activators which would restore sensitivity. However this prospect does not seem to be generally applicable and each cell type may have to be considered separately. In colon cancer cells PKC activation has been shown to be responsible for, or to contribute to, intrinsic drug resistance (Dong *et al.*, 1991). Multi-drug resistant human KB and mouse sarcoma 180 cells contained significantly higher levels of PKC-α than their drug-sensitive counterparts (Posada *et al.*, 1989) and inhibition of endogenous PKC activity in the insensitive cells did not maintain or increase but decreased resistance to adriamycin. Elevated PKC activity in cells as a cause of decreased rather than increased sensitivity to antitumour cytotoxicants has been explained by PKC-mediated activation of gp 170, the product of the multi-drug resistance gene *MDR1* (Ford & Hait, 1990). Adriamycin-resistant HL-60 cells contained the same level of PKC-α but less PKC-β than their sensitive counterparts (Aquino *et al.*, 1990). Moreover they also possessed PKC-γ, which was absent in adriamycin-sensitive HL-60 cells. These results underline the functional importance of specific PKC isoforms in contrast to overall enzyme levels.

### Regulation of PKC

Is specificity possible at the level of PKC regulation? Mechanisms of PKC regulation are complex, but some are amenable to interference by exogenous agents. Exposure of cells to $Ca^{2+}$ or phorbol esters leads to the redistribution of PKC from the cellular cytosol to the particulate subcellular fraction (Kraft & Anderson, 1983). This association with membranes is presumably normally mediated by diacylglycerol and brings PKC into close proximity with its cofactor phospholipid. Some selectivity was observed in $Ca^{2+}$-mediated PKC redistribution in rat pituitary cells (Kiley *et al.*, 1990). An increase in cytosolic $Ca^{2+}$ caused transcloation of PKC-α and -β but not of PKC-ε. Changes in PKC localisation probably involve its binding to specific 'receptors for activated C-kinase' (RACKs), which were proposed to exist at multiple intracellular sites and the plasma membrane

(Mochley-Rosen *et al.*, 1991). The PKC downregulation observed on sustained exposure to PKC activating ligands is probably due to an increased proteolytic degradation of PKC by endogenous protease (Young *et al.*, 1987; Pontremoli *et al.*, 1990). Differential kinetics of depletion of PKC isozymes have been reported in several cellular systems (Huang *et al.*, 1989; Schaap *et al.*, 1990; Ase *et al.*, 1988; Adams & Gullick, 1989), indicating that the rate at which PKC is downregulated is isoform- and cell type-specific. In rat pituitary cells phorbol ester treatment caused a total loss of immunoreactive PKC-β and PKC-ε, but only partial downregulation of PKC-α (Kiley *et al.*, 1990), which suggests that prolonged exposure to phorbol esters might be a means to generate a cell population containing mainly PKC-α. To complicate matters downregulation of PKC-ε, but not of PKC-β, by TPA in thymocytes depended on elevated levels of $Ca^{2+}$ (Strulovici *et al.*, 1991). The rate at which PKC recovers after long-term phorbol ester treatment is also isozyme-dependent (Huwiler *et al.*, 1991). In contrast to the original hypothesis (Kraft & Anderson, 1983) events which cause PKC redistribution and down-regulation, respectively, are probably not a prelude to, or a corollary of, enzyme activation (Trilivas *et al.*, 1991; Pears & Parker; 1991; Lindner *et al.*, 1991; Bradshaw *et al.*, 1992). Therefore it is conceivable that agents will eventually be found which affect PKC by interference with enzyme redistribution, binding to RACKs, or enzyme downregulation, without being enzyme activators. This possibility is particularly appealing in view of the finding that downregulation of PKC can elicit cytotoxicity in certain cell types. TPA at concentrations provoking PKC down-regulation killed neoplastic thyroid follicular cells which express a mutant *ras* oncogene, but not normal follicular cells (Bond *et al.*, 1992).

That PKC isoenzymes can be regulated independently has been shown in human leukaemia cell lines. When Jurkat leukaemic T cells were grown continuously in the presence of phorbol esters they exhibited a 6-fold decrease in enzymatically active cytosolic PKC (Isakov *et al.*, 1990). This decrease was selective for PKC-α, whereas PKC-β or γ were not affected. The expression of the corresponding genes was not changed. These results contrast with experiments in which the PKC phenotype was compared between HL-60 cells and a subline PR-17 with acquired resistance to the differentiating and cytostatic properties of TPA (McSwine-Kennick *et al.*, 1991). Crude preparations of the resistant cells contained only 70% of PKC activity in wild type HL-60 cells, but other aspects of the PKC phenotype were equivalent, among them the relative expression of the PKC-α and -β proteins. Both isozymes were downregulated similarly in either cell line on exposure to TPA. However in the wild-type cells steady state levels and transcriptional rates of PKC-β mRNA were increased between 3 to 5-fold after incubation with TPA for 1 to 2 days; similarly PKC-β gene transcription was increased, whereas PKC-α mRNA was not affected. These changes did not occur in the TPA-resistant PR-17 cells. This work suggests that specific alterations in the expression of the PKC-β gene (or of other genes regulated by activated PKC isozymes) are important for the induction of monocytic differentiation in HL-60 cells.

Molecular events which regulate PKC isozyme gene expression seem to be part of a complex hierarchy. This was shown in NCI H209 human small cell lung cancer cells in which co-insertion of a human c-*myc* and a viral Ha-*ras* gene were associated with distinct changes in expression of PKC-α and -β, both at the transcript and protein levels (Barr *et al.*, 1991). These changes accompanied the adoption by the cells of morphological properties of large cell carcinoma. In naive NCI H209 cells PKC-α was the dominant PKC species. Expression of the *myc* gene, but not of the *ras* gene, caused a 5- to 10-fold increase in the PKC-β isoform transcript and protein. Coinsertion of *ras* and *myc* reversed the increased PKC-β transcript levels induced by *myc* alone and also elicited the redistribution of PKC-β protein from the cytosol to the membrane and a decrease in membrane-associated PKC-α.

**Bryostatins**

The byrostatins are macrocyclic lactones isolated from marine bryozoans, of which 17 different derivatives have been described (Pettit, 1991). They activate PKC, in some cells as potently as TPA, in others somewhat less strongly (Berkow & Kraft, 1985; Smith *et al.*, 1985), and bryostatin 1 was able to displace phorbol esters from their receptors in a semi-purified rat brain PKC preparation at subnanomolar concentrations (De Vries *et al.*, 1988). A weak structural similarity exists between the bryostatin and phorbol ester molecules as revealed by computer modelling of the spatial arrangement of sets of several oxygen atoms (Wender *et al.*, 1988). The current clinical trials of bryostatin 1 have been initiated mainly on the basis of its interesting antineoplastic activity in rodent models. Inhibition of cell proliferation by bryostatins has been demonstrated *in vitro* in a variety of murine tumour cell lines traditionally used as antitumor drug screens (Pettit *et al.*, 1982), also in M5076 reticulum cell sarcoma, B16 melanoma and L10A B-cell lymphoma (Hornung *et al.*, 1992), and in human chronic myelomonocytic leukaemia cells (Lilly *et al.*, 1991). They have displayed antitumour activity *in vivo* on multiple i.p. administration in mice with P388 lymphocytic leukaemia, ovarian sarcoma (Pettit *et al.*, 1970), B16 melanoma (Schuchter *et al.*, 1991), M5076 sarcoma and L10A lymphoma (Hornung *et al.*, 1992). Significantly, TPA was not cytostatic in the B16 melanoma model. Bryostatins are potent immunomodulators, they activate T-cells, augment neutrophil- and monocyte-mediated cytotoxicity and stimulate bone marrow progenitor cells (Hess *et al.*, 1988; May *et al.*, 1987). A truly puzzling feature of their biological activity is its paradoxical nature. In many cell lines they are not only agonistic with tumour-promoting phorbol esters but also able to antagonise biochemical responses elicited by themselves or by phorbol esters. They mimic TPA in that they inhibited phorbol ester binding and caused mitogenesis in Swiss 3T3 cells (Smith *et al.*, 1985), stimulated human polymorphonuclear leukocytes (Berkow & Kraft, 1985), and induced ornithine decarboxylase, while inhibiting cell-cell communication in keratinocytes (Sako *et al.*, 1987; Pasti *et al.*, 1988). They also inhibited the growth of A549 cells, albeit only for a period of 24 h, beyond which growth inhibition was slowly reversed (Dale & Gescher, 1989). Bryostatin 1 failed to induce terminal differentiation of human colon cancer (McBain *et al.*, 1988) and of HL-60 cells (Kraft *et al.*, 1986). Nevertheless morphological or functional evidence of incomplete differentiation was noticed in most of these lines on exposure to bryostatin. In contrast, bryostatins blocked the differentiation induced by phorbol esters in HL-60 (Kraft *et al.*, 1986), human colon cancer (McBain *et al.*, 1988) and primary mouse epidermal cells (Sako *et al.*, 1987). They also restored the differentiation response in Friend erythroleukaemia cells, in which drug-induced differentiation was suppressed by phorbol esters (Dell'Aquila *et al.*, 1987). Bryostatin 1 blocked both the inhibitory effects on growth elicited by lower concentrations of itself or by TPA (Dale & Gescher, 1989). Similarly, when combined with TPA bryostatin prevented the TPA-induced inhibition of proliferation of $GH_4C_1$ in rat pituitary tumour cells (Mackanos *et al.*, 1991). Relevant to the prospective use of bryostatin 1 in the clinic is the finding that it lacked tumour-promoting properties in the SENCAR mouse model, indeed it inhibited TPA-induced tumour promotion (Hennings *et al.*, 1987). The conclusion to be drawn from the perplexing literature on bryostatins is that they exert some of their effects *via* mechanisms which are independent of those operated by phorbol esters. In none of the above examples was there any evidence that the paradoxical effects were accompanied by paradoxical consequences on PKC redistribution or downregulation (Dale *et al.*, 1989). Therefore the ability of the byrostatins to antagonise certain phorbol ester effects suggests that they interfere with events downstream of PKC activation. Alternatively certain isozymes like PKC-ζ or other cellular signal transduction mechanisms not activated by phorbol esters may be involved. Studies of the phosphorylation pattern in cells exposed to either bryostatins or phorbol esters are consistent with this hypothesis. In HL-60 cells bryostatin 1 caused the phosphorylation of two 70 kDa proteins, perhaps of cytoskeletal origin, in addition to those which were phosphorylated in the presence of phorbol dibutyrate (Warren *et al.*, 1988). The bryostatin-specific response could also be evoked by concentrations of phorbol dibutyrate which exceeded that necessary to induce differentiation by a factor of 100. This finding underlines the possibility that bryostatins and high concentrations of phorbol esters affect specific PKC isoforms differently from phorbol esters at low concentrations. Bryostatin 1 was less efficient than TPA in competing for specific phorbol ester receptor sites of isolated PKC-$\beta$, whereas it equalled TPA in its ability to bind to PKC-$\alpha$ and -$\gamma$ (Kraft *et al.*, 1988). A significant difference between bryostatins and TPA may be the direction of PKC redistribution. Treatment of HL-60 cells with bryostatin 1 led to the specific redistribution of activated PKC-$\beta_{II}$, but not of PKC-$\alpha$ to the nuclear envelope, where it triggered the phosphorylation of the polypeptide lamin B (Fields *et al.*, 1988). In contrast, phorbol dibutyrate caused PKC redistribution towards the plasma membrane (Fields *et al.*, 1988). Such a discrepancy in PKC relocalisation has yet to be reported for cell lines other than HL-60, in which the bryostatins and phorbol esters possess antagonistic properties.

**Table I** PKC inhibitors

| | |
|---|---|
| A. at the catalytic domain | |
| Staurosporine | (Tamaoki *et al.*, 1986) |
| Hydroxystaurosporine UCN-01 | (Akinage *et al.*, 1991) |
| N-Benzoylstaurosporine CGP 41251 | (Meyer *et al.*, 1989) |
| K252a | (Kase *et al.*, 1987) |
| H-7 | (Hidaka *et al.*, 1984) |
| Aminoacridine | (Hannun & Bell, 1988) |
| Sangivamycin | (Lomis & Bell, 1988) |
| Chelerythrine | (Herbert *et al.*, 1990) |
| Suramin | (Mahoney *et al.*, 1990) |
| Dequalinium | (Rotenberg *et al.*, 1990) |
| Protein PKCI-1 | (Pearson *et al.*, 1990) |
| B. at the regulatory domain: | |
| Calphostin C | (Kobayashi *et al.*, 1989) |
| Chlorpromazine | (Mori *et al.*, 1983) |
| Trifluoperazine | (Wise & Kuo, 1983) |
| Ether lipids | (Marx *et al.*, 1988) |
| Hexadecylphosphocholine | (Überall *et al.*, 1991*a*; Geilen *et al.*, 1991) |
| Tamoxifen | (O'Brian *et al.*, 1985) |
| Sphingosine | (Hannun & Bell, 1987) |
| Trimethylsphingosine | (Endo *et al.*, 1991) |
| Adriamycin | (Hannun *et al.*, 1989) |
| (*R*)-Niguldipine | (Überall *et al.*, 1991*b*) |
| NPC 15437 | (Sullivan *et al.*, 1991) |

### PKC inhibitors and growth arrest

In view of the role of PKC in the mediation of mitogenic signals the concept of inhibiting rather than activating PKC appears to be the more logical strategy in the search for novel avenues in cancer treatment. In support of this idea aberrant expression of PKC has been associated with certain malignancies and metastasis. Transfection of a mutant PKC-$\alpha$ gene into BALB/c 3T3 fibroblasts enhanced their tumourigenicity (Megidish & Mazurek, 1989), but this finding has subsequently been disputed (Borner *et al.*, 1991). Metastatic potential has also been correlated with overexpression of normal PKC (Persons *et al.*, 1988; Housey *et al.*, 1988).

Which are the sites within PKC which could be considered as targets for the design of inhibitors, and which degree of selectivity do they allow? There are firstly the regulatory domain, which contains the activator binding site, secondly the ATP binding site on the catalytic domain, and thirdly the substrate binding domain. *A priori* the chance of selectivity seems to be lowest in the case of inhibitors of the ATP binding site, as it shows strong homology with other serine and tyrosine kinases. Specificity seems more likely for agents which inhibit predominantly at the regulatory site, and even more possible in the case of inhibitors of the substrate binding site. Examples of agents which inhibit PKC by interaction with the regulatory or catalytic domains are listed in Table I. On the whole their specificity for PKC is moderate. Therefore it is unclear whether their cytostatic potential is mediated *via* PKC, other kinases or a combination. Important in the context of this discussion is that potent PKC inhibitors do possess growth-inhibitory or cytotoxic properties (Tamaoki & Nakano, 1990). Thus properties of two representative molecules, staurosporine and sphingosine (Figure 2), are briefly described in the following. Staurosporine, an indole carbazole, inhibits at the catalytic domain and is one of the most potent PKC inhibitors yet described (Tamaoki *et al.*, 1986), and sphingolipids are natural constituents of cells and inhibit the regulatory domain (Hannun & Bell, 1989). Staurosporine showed an effect on tumour cell invasion and is thus of potential therapeutic interest. At non-toxic concentrations it antagonised the invasion of human bladder carcinoma cells through an artificial basement membrane by inhibiting cell motility (Schwartz *et al.*, 1990). The drug displayed an unexpected element of specificity in that it affected cell cycle progression and nuclear morphology differently in MOLT-4 human lymphocytic leukaemia cells as compared to normal human lymphocytes (Bruno *et al.*, 1992). Minor structural modifications have yielded derivatives with greater selectivity for PKC. N-Benzoylstaurosporine CGP 41251 (Figure 2) is a less potent but more selective PKC inhibitor than staurosporine (Meyer *et al.*, 1989). Both agents had comparable antitumour activity against a human bladder carcinoma xenograft grown in nude mice. Likewise RO 31-8220 and RO 31-7549 (Figure 2), cogeners of the staurosporine aglycone, were more specific PKC inhibitors than staurosporine *in vitro* (Davis *et al.*, 1989) and in intact cells (Dieter & Fitzke, 1991). UCN-01, hydroxystaurosporine (Figure 2), is a more selective but weaker and less cytotoxic PKC-inhibitor than staurosporine. But unlike staurosporine, it displayed antineoplastic activity against three human tumour xenografts in nude mice and two murine models, all of which possessed certain aberrations in cellular signal transduction (Akinaga *et al.*, 1991). As is the case with the bryostatins, staurosporine displays paradoxical effects in that it not only inhibits but also mimics certain phorbol ester effects. Staurosporine enhanced TPA-induced responses in mouse keratinocytes, such as cellular maturation (Dlugosz & Yuspa, 1991). It was also a weak tumour promoter in the CD-1 mouse model, even though it inhibited promotion induced by phorbol esters (Yoshizawa *et al.*, 1990). There is an indication that inhibitors of the staurosporine type do not repress all PKC isoforms equally, as a $Ca^{2+}$-independent PKC isoform from porcine spleen was unaffected by the staurosporine analogue K252a (Gschwendt *et al.*, 1989).

Lysophingolipids such as sphingosine (Figure 2) are metabolites of membrane sphingolipids which may be cellular second messengers (Hannun & Bell, 1989). Sphingosine inhibited the TPA-induced differentiation of HL-60 cells (Merrill *et al.*, 1989), and dihydrosphingosine in the $10^{-6}$ M concentration range exerted cytostasis and cytotoxicity against Chinese hamster ovary cells (Stevens *et al.*, 1990). Both effects were mechanistically attributed to PKC inhibition, which seems to require pH conditions under which the sphingosine amine function is protonated (Bottega *et al.*, 1989). Unlike sphingosine itself, its synthetic analogues N,N-dimethyl- and N,N,N-trimethyl-sphingosine (Figure 2) had moderate antineoplastic activity *in vivo* against a human gastric carcinoma cell line grown in nude mice (Endo *et al.*, 1991), and the latter also inhibited the metastatic potential of murine B16 melanoma cells *in vivo* (Okoshi *et al.*, 1991). Of the anticancer drugs in clinical use which possess PKC-inhibitory properties (Table I) the most remarkable is tamoxifen. This drug and its metabolites interact with the

**Figure 2** Structures of PKC inhibitors.

regulatory domain of PKC and inhibit the enzyme at pharmacologically relevant concentrations in the $10^{-5}$ M range (O Brian *et al.*, 1985). PKC inhibition might contribute to its mechanism of antineoplastic action.

The regulatory domain of PKC contains a sequence with a cluster of basic residues, which resembles PKC phosphorylation sites, except that the potential phosphate acceptor serine is replaced by alanine (House & Kemp, 1987). Binding of this 'pseudosubstrate' to the substrate binding domain is thought to be responsible for the maintenance of inactive PKC in the absence of an activating molecule. A synthetic peptide corresponding to this sequence, Arg-Phe-Ala-Arg-Lys-Gly-Ala-Leu-Arg-Gln-Lys-Asn-Val, turned out to be an inhibitor of exquisite specificity for PKC in isolated enzyme preparations (House *et al.*, 1987) and permeabilised cells (Eichholtz *et al.*, 1990). Manipulations to achieve intracellular delivery of this or similar pseudosubstrates and to restrain their proteolytic destruction might furnish exciting molecules with a better chance of specificity than that displayed by the compounds shown in Table I.

None of the PKC inhibitors so far discovered are particularly selective with respect to PKC isozymes. Nevertheless isozyme-specificity was demonstrated when PKC was irreversibly destroyed by acidic phospholipids in incubates lacking divalent cations (Huang & Huang, 1990; Pelosin *et al.*, 1990). Of the three major PKC isozymes, PKC-γ was the most and PKC-α the least susceptible to this inactivating effect. As it seems to depend on rather artificial *in vitro* conditions it is unlikely to be exploitable in intact cells.

A further example of the complex nature of pharmacological modulation of PKC activity is the activity of the experimental antineoplastic alkyllysophospholipid ET-18-$OCH_3$ (Figure 2), a synthetic analogue of 2-lysophosphatidylcholine. ET-18-$OCH_3$ inhibited PKC purified from HL-60 cells with a $K_i$ of 9–15μM (Zheng *et al.*, 1990), and the inhibition was competitive with respect to phosphatidylserine. In contrast, the drug activated PKC instead of inhibiting it when the enzyme was prepared from HL-60 cells such that it was still associated with the plasma membrane (Heesbeen *et al.*, 1991).

### Conclusions

Because of its pivotal importance for signal transduction mechanisms PKC is undoubtedly a logical target for drug intervention. Subtle differences in terms of levels and heterogeneity of PKC between normal and neoplastic tissues and between different neoplasias are beginning to emerge. These differences might provide selective targets for rational drug design. Among the PKC isozymes the β-form seems to play a crucial role in differentiation, and the design of differentiation inducers should perhaps target PKC-β. In *ras*-transformed malignancies PKC-α might mediate mitogenic and PKC-ε anti-mitogenic events. The selective physiological roles of these and other PKC subforms will undoubtedly become clearer soon. Pharmacological alteration of the balance between different PKC isoforms might turn out to be as critical for cell dynamics as modification of a specific PKC isoform.

The nature and extent of effects of some of the cytostatic PKC modulators described above are paradoxical and confusing. This might be related to the fact that the functional consequence of PKC modulation seems to be different for each cell type and that the specific roles of PKC isozymes might be cell type-specific. It appears that in order to be of therapeutic value PKC modulators need to be more selective than the currently known agents. Details of the mechanistic link between the growth-inhibitory properties of these agents and the extent of their ability to modulate PKC are still essentially unknown. Bryostatin 1 activates PKC but also antagonises phorbol esters; staurosporine is a potent inhibitor but also exerts phorbol ester-agonistic effects; ET-18-$OCH_3$ is a PKC inhibitor, but under certain conditions an activator. They all mobilise mechanisms yet to be understood in addition to gross PKC modulation. Nevertheless selective pharmacological intervention with PKC-related process might eventually be possible, as isozymes selectivity among PKC activators seems to be no longer an elusive goal. The phorbol esters sapintoxin A and DOPPA are 'lead compounds' in this respect. Inhibitors like staurosporine lack selectivity, but chemical modification has already furnished molecules with increased selectivity for PKC and increased experimental antineoplastic properties compared to the parent molecule. PKC pseudosubstrate analogues might offer even better specificity.

There is no doubt that the complicated interaction between different second messenger signalling systems which involve cascades of kinases, one of which is PKC, will render selective drug action using PKC modulators difficult. Whether it is too complex for therapeutic intervention remains to be seen.

### Note added in proof

'In MCF-7 cells, the growth of which is inhibited by TPA but only marginally by bryostatin 1, cytosolic PKC-α was down-regulated by bryostatin 1 without any observable redistribution to the membrane, whereas TPA caused PKC-α redistribution before it was down-regulated (Kennedy *et al.*, 1992). A cogent model which describes common structural features of PKC activators including the bryostatins has very recently been described (Rando & Kishi, 1992)'.

I thank the CRC for generous financial support of the work on PKC activators (project grant SP1518), Drs G. Brooks and J. Lord for constructive criticism of the manuscript, and Dr T.D. Bradshaw and Ms C. Stanwell for helpful comments.

### References

ADAMS, J.C. & GULLICK, W.J. (1989). Differences in phorbol ester-induced downregulation of protein kinase C between cell lines. *Biochem. J.*, **257**, 905–911.

AKINAGA, S., GOMI, K., MORIMOTO, M., TAMAOKI, T. & OKABE, M. (1991). Antitumor activity of UCN-01, a selective inhibitor of protein kinase C, in murine and human tumor models. *Cancer Res.*, **51**, 4888–4892.

AKITA, Y., OHNO, S., KONNO, Y., YANO, A. & SUZUKI, K. (1990). Expression and properties of two distinct classes of the phorbol ester receptor family, four conventional protein kinase C types, and a novel protein kinase C. *J. Biol. Chem.*, **265**, 354–362.

AQUINO, A., WARREN, B.S., OMICHINSKY, J., HARTMAN, K.D. & GLAZER, R.I. (1990). Protein kinase C-γ is present in adriamycin resistant HL-60 leukemia cells. *Biochem. Biophys. Res. Commun.*, **166**, 723–728.

ASE, K., BERRY, N., KIKKAWA, U., KISHIMOTO, A. & NISHIZUKA, Y. (1988). Differential down-regulation of protein kinase C subspecies in KM3 cells. *FEBBS Lett.*, **236**, 396–400.

BARR, L.F., MABRY, M., NELKIN, B.D., TYLER, G., MAY, W.S. & BAYLIN, S.B. (1991). *c-myc* Gene-induced alterations in protein kinase C expression: a possible mechanism facilitating *myc-ras* gene complementation. *Cancer Res.*, **51**, 5514–5519.

BASU, A., KOZIKOWSKI, A.P., SATO, K. & LAZO, J.S. (1991). Cellular sensitization to cis-diamminedichloroplatinum(II) by novel analogues of the protein kinase C activator lyngbyatoxin A. *Cancer Res.*, **51**, 2511–2514.

BASU, A., TEICHER, B.A. & LAZO, J.S. (1990). Involvement of protein kinase C in phorbol ester-induced sensitization of HeLa cells to cis-diamminedichloroplatinum(II). *J. Biol. Chem.*, **265**, 8451–8457.

BECKER, D., BEEBE, S.J. & HERLYN, M. (1990). Differential expression of protein kinase C and cAMP-dependent protein kinase in normal human melanocytes and malignant melanomas. *Oncogene*, **5**, 1133–1139.

BECKWITH, M., LONGO, D.L., O'CONNELL, C.D., MORATZ, C.M. & URBA, W.J. (1990). Phorbol ester-induced cell-cycle-specific growth inhibition of human B-lymphoma cells. *J. Natl Cancer Inst.*, **82**, 501–509.

BEH, I., SCHMIDT, R. & HECKER, E. (1989). Two isozymes of PKC found in HL-60 cells show a difference in activation by the phorbol ester TPA. *FEBS Lett.*, **249**, 264–266.

BERKOW, R.L. & KRAFT, A.S. (1985). Bryostatin, a non-phorbol macrocyclic lactone, activates human polymorphonuclear leukocytes and binds to the phorbol ester receptor. *Biochem. Biophys. Res. Commun.*, **131**, 1109–1116.

BLUMBERG, P.M. (1991). Complexities of the protein kinase C pathway. *Molec. Carcinogenesis*, **4**, 339–344.

BOND, J.A., DAWSON, T., LEMOINE, N.R. & WYNFORD-THOMAS, D. (1992). Effect of serum growth factors and phorbol ester on growth and survival of human thyroid epithelial cells expressing mutant *ras*. *Molec. Carcinogenesis* (in press).

BORNER, C., NICHOLS GUADAGNO, S., HSIEH, L.-L., HSIAO, W.-L.W. & WEINSTEIN, I.B. (1990). Transformation by a *ras* oncogene causes increased expression of protein kinase C-α and decreased expression of protein kinase C-ε. *Cell Growth & Diff.*, **1**, 653–660.

BORNER, C., FILIPUZZI, I., WEINSTEIN, I.B. & IMBER, L. (1991). Failure of wild-type or a mutant form of protein kinase C-α to transform fibroblasts. *Nature*, **353**, 78–83.

BOTTEGA, R., EPAND, R.M. & BALL, E.H. (1989). Inhibition of protein kinase C by sphingosine correlates with the presence of positive charge. *Biochem. Biophys. Res. Commun.*, **164**, 102–107.

BRADSHAW, T.D., GESCHER, A. & PETTIT, G.R. (1991). The effects of fetal calf serum on growth arrest caused by activators of protein kinase C. *Int. J. Cancer.*, **47**, 929–932.

BRADSHAW, T.D., GESCHER, A. & PETTIT, G.R. (1992). Modulation by staurosporine of phorbol ester-induced effects on growth and protein kinase C localisation in A549 human lung carcinoma cells. *Int. J. Cancer* (in press).

BROOKS, G., BIRCH, M. & HART, I. (1990). Effects of biologically active tumour-promoting and non-promoting phorbol esters on *in vitro* growth of melanocytic cells. *Pigm. Cell. Res.*, **3**, 98–100.

BROOKS, G., EVANS, A.T., AITKEN, A. & EVANS, F.J. (1989). Tumour-promoting and hyperplastic effects of phorbol and daphnane esters in CD-1 mouse skin and a synergistic effect of calcium ionophore with the nonpromoting activator of protein kinase C, sapintoxin A. *Carcinogenesis*, **10**, 283–288.

BROOKS, G., WILSON, R.E., DOOLEY, T.P., GOSS, M.W. & HART, I.R. (1991). Protein kinase C down-regulation, and not transient activation, correlates with melanocyte growth. *Cancer Res.*, **51**, 3281–3288.

BRUNO, S., ARDELT, B., SKIERSKI, J.,S., TRAGANOS, F. & DARZYNKIEWICZ, Z. (1992). Different effects of staurosporine, an inhibitor of protein kinases, on the cell cycle and chromatin structure of normal an leukemic lymphocytes. *Cancer Res.*, **51**, 470–473.

CHOI, P.M., TCHOU-WONG, K.-M. & WEINSTEIN, I.B. (1990). Overexpression of protein kinase C in HT29 colon cancer cells causes growth inhibition and tumor suppression. *Molec. Cell. Biol.*, **10**, 4650–4657.

CHOI, P.M. & WEINSTEIN, I.B. (1991). The modulation of growth by HMBA in PKC overproducing HT29 colon cancer cells. *Biochem. Biophys. Res. Comm.*, **181**, 809–817.

DALE, I.L., BRADSHAW, T.D., GESCHER, A. & PETTIT, G.R. (1989). Comparison of effects of bryostatins 1 and 2 and 12-*O*-tetradecanoylphorbol-13-acetate on protein kinase C activity A549 human lung carcinoma cells. *Cancer Res.*, **49**, 3242–3245.

DALE, I.L. & GESCHER, A. (1989). Effects of activators of protein kinase C, including bryostatins 1 and 2, on the growth of A549 human lung carcinoma cells. *Int. J. Cancer*, **43**, 158–163.

DAVID, P.D., HILL, C.H., KEECH, E., LAWTON, G., NIXON, J.S., SEDGWICK, A.D., WADSWORTH, J., WESTMACOTT, D. & WILKINSON, S.E. (1989). Potent selective inhibitors of protein kinase C. *FEBS Lett.*, **259**, 61–63.

DELL'AQUILA, M.L., NGUYEN, H.T., HERALD, C.L., PETTIT, G.R. & BLUMBERG, P.M. (1987). Inhibition by bryostatin 1 of the phorbol ester-induced blockade of differentiation in hexamethylene bisacetamide-treated friend erythroleukemia cells. *Cancer Res.*, **47**, 6006–6009.

DE VRIES, D.J., HERALD, C.L., PETTIT, G.R. & BLUMBERG, P.M. (1988). Demonstration of sub-nanomolar affinity of bryostatin 1 for the phorbol ester receptor in rat brain. *Biochem. Pharmacol.*, **37**, 4069–4073.

DIETER, P. & FITZKE, E. (1991). RO 31-8220 and RO 3107549 shows improved selectitity for protein kinase C over staurosporine in macrophages. *Biochem. Biophys. Res. Commun.*, **181**, 396–401.

DONG, Z., WARD, N.E., FAN, D., GUPTA, K.P. & O'BRIAN, C.A. (1991). *In vitro* model for intrinsic drug resistance: effects of protein kinase C activators on the chemosensitivity of cultured human colon cancer cells. *Molec. Pharmacol.*, **39**, 563–569.

DLUGOSZ, A.A. & YUSPA, S.H. (1991). Staurosporine induces protein kinase C agonist effects and maturation of normal and neoplastic mouse keratinocytes *in vitro*. *Cancer Res.*, **51**, 4677–4684.

EICHHOLTZ, T., ALBLAS, J. VAN OVERVELD, M., MOOLENAAR, W. & PLOEGH, H. (1990). A pseudosubstrate peptide inhibits protein kinase C-mediated phosphorylation in permeabilized Rat-1 cells. *FEBS Lett.*, **261**, 147–150.

ENDO, K., IGARASHI, Y., NISAR, M., ZHOU, Q. & HAKOMORI, S. (1991). Cell membrane signalling as target in cancer therapy: inhibitory effect of N,N-dimethyl and N,N,N-trimethyl sphingosine derivatives on *in vitro* and *in vivo* growth of human tumor cells in nude mice. *Cancer Res.*, **51**, 1613–1618.

FIELDS, A.P., PETTIT, G.R. & MAY, W.S. (1988). Phosphorylation of lamin B at the nuclear membrane by activated protien kinase C. *J. Biol. Chem.*, **263**, 8253–8260.

FORD, J.M. & HAIT, W.N. (1990). Pharmacology of drugs that alter multidrug resistance in cancer. *Pharmacol. Rev.*, **42**, 155–199.

GEILEN, C.C., HAASE, R., BUCHNER, K., WIEDER, T., HUCHO, F. & REUTER, W. (1991). The phospholipid analogue, hexadecylphosphocholine, inhibits protein kinase C *in vitro* and antagonises phorbol ester-stimulated cell proliferation. *Eur. J. Cancer*, **27**, 1650–1653.

GESCHER, A. (1985). Antiproliferative propteries of phorbol ester tumour promoters. *Biochem. Pharmacol.*, **34**, 2587–2592.

GSCHWENDT, M., LEIBERSPERGER, H. & MARKS, F. (1989). Differentiative action of KJ252a on protein kinase C and a calcium-unresponsive, phorbol ester/phospholipid-activated protein kinase. *Biochem. Biophys. Res. Commun.*, **164**, 974–982.

HANNUN, Y.A. & BELL, R.M. (1987). Lysophingolipids inhibit protein kinase C: implications for sphingolipidoses. *Science*, **235**, 670–674.

HANNUN, Y.A. & BELL, R.M. (1988). Aminoacridines, potent inhibitors of protein kinase C. *J. Biol. Chem.*, **263**, 5124–5131.

HANNUN, Y.A. & BELL, R.M. (1989). Functions of sphingolipids and sphingolipid breakdown products in cellular regulation. *Science*, **243**, 500–507.

HANNUN, Y.A., FOGLESONG, R.J. & BELL, R.M. (1989). The adriamycin-iron (III) complex is a potent inhibitor of protein kinase C. *J. Biol. Chem.*, **264**, 9960–9966.

HASS, R., PFANNKUCHE, H.J., KHARBANDA, S., GUNJI, H., MEYER, G., HARTMAN, A., HIDAKA, H., RESCH, K., KUFE, D. & GOPPELT-STRÜBE, M. (1991). Protein kinase C activation and protooncogene expression in differentiation/retrodifferentiation of human U-937 leukemia cells. *Cell Growth & Diff.*, **2**, 541–548.

HECKER, E. (1985). Cell membrane associated protein kinase C as receptor of tumour promoters and the phenotypic expression of tumours. *Arzneim. Forsch. (Drug Res.)*, **35**, 1890–1899.

HEESBEEN, E.C., VERDONCK, L.F., HERMANS, S.W.G., VAN HEUGTEN, H.G., STAAL, G.E.J. & RIJKSEN, G. (1991). Alkyllysophospholipid ET-18-$OCH_3$ acts as an activator of protein kinase C in HL-60 cells. *FEBS Let.*, **290**, 231–234.

HENNINGS, H., BLUMBERG, P.M., PETTIT, G.R., HERALD, C.L., SHORES, R. & YUSPA, S. (1987). Bryostatin 1, an activator of protein kinase C, inhibits tumor promotion by phorbol esters in Sencar mouse skin. *Carcinogenesis*, **9**, 1343–1346.

HERBERT, J.M., AUGEREAU, J.M., GLEYE, J. & MAFFRAND, J.P. (1990). Cherlerythrine is a potent and specific inhibitor of protein kinase C. *Biochem. Biophys. Res. Commun.*, **172**, 993–999.

HESS, A.D., SILANSKIS, M.K., ESA, A.H., PETTIT, G.R. & MAY, W.S. (1988). Activation of human T lymphocytes by bryostatin 1. *J. Immunol.*, **141**, 3263–3269

HIDAKA, H., INAGAKI, M., KAWAMOTO, S. & SASAKI, Y. (1984). 1-(5-Isoquinolinesulfonyl)-2-methylpeperazine (H7), a potent inhibitor of protein kinase C (PKC). *Biochemistry*, **23**, 5036–5041.

HIRAI, M., GAMOU, S., KOBAYASHI, M. & SHIMIZU, N. (1989). Lung cancer cells often express high levels of protein kinase C activity. *Jpn. J. Cancer Res.*, **80**, 204–208.

HOCEVAR, B.A. & FIELDS, A.B. (1991). Selective translocation of $\beta_{II}$-protein kinase C to the nucleus of human promyelocytic (HL-60) leukemia cells. *J. Biol. Chem.*, **266**, 28–33.

HOOPER, W.C., ABRAHAM, R.T., ASHENDEL, C.L. & WOLOSCHAK, G.E. (1989). Differential responsiveness to differential expression of protein kinase C in KG-1 and KG-1a human myeloid leukemia cells. *Biochem. Biophys. Acta*, **1013**, 47–54.

HORNUNG, R.L., PEARSON, J.W., BECKWITH, M. & LONGO, D.L. (1992). Preclinical evaluation of bryostatin as an anticancer agent against several murine tumor cell lines. *In vitro vs in vivo* activity. *Cancer Res.*, **52**, 101–107.

HOUSE, C. & KEMP, B.E. (1987). Protein kinase C contains a pseudosubstrate prototype in its regulatory domain. *Science*, **238**, 1726–1728.

HOUSE, C., WETTENHALL, R.E.H. & KEMP, B.E. (1987). The influence of basic residues on the substrate specificity of protein kinase C. *J. Biol. Chem.*, **262**, 772–777.

HOUSEY, G.M., JOHNSON, M.D., HSIAS, W.L.W., O'BRIAN, C.A., MURPHY, J.P., KIRSCHMEIER, P. & WEINSTEIN, I.B. (1988). Overproduction of protein kinase C causes disordered growth control in rat fibroblasts. *Cell*, **52**, 343–354.

HUANG, F.L., YOSHIDA, Y., CUNHA-MELO, J.R., BEAVEN, M.A. & HUANG, K.P. (1989). Differential down-regulation of protein kinase C isozymes. *J. Biol. Chem.*, **264**, 4238–4243.

HUANG, K.P. & HUANG, F.L. (1990). Differential sensitivity of protein kinase C isozymes to phospholipid–induced inactivation. *J. Biol. Chem.*, **265**, 738–744.

HUWILER, A., FABBRO, D. & PFEILSCHIFTER, J. (1991). Differential recovery of protein kinase C-α and -ε isozymes after long-term phorbol ester treatment in rat renal mesangial cells. *Biochem. Biophys. Res. Commun.*, **180**, 1422–1428.

ISAKOV, N., MCMAHON, P. & ALTMAN, A. (1990). Selective post-transcriptional down-regulation of protein kinase C isoenzymes in leukemic T cells chronically treated with phorbol ester. *J. Biol. Chem.*, **265**, 2091–2097.

ISONISHI, S., ANDREWS, P.A. & HOWELL, S.B. (1990). Increased sensitivity to *cis*-diamminedichloroplatinum(II) in human ovarian carcinoma cells in response to tretment with 12-*O*-tertradecanoylphorbol 13-acetate. *J. Biol. Chem.*, **265**, 3623–3627.

KASE, H., IWAHASHI, K., NAKANISHI, S., MATSUDA, Y., YAMADA, K., TAKAHASHI, M., MURAKATA, C., SATO, A. & KANEKO, M. (1987). K-252 compounds, novel and potent inhibitors of protein kinase C and cyclic nucleotide-dependent protein kinases. *Biochem. Biophys. Res. Commun.*, **142**, 436–440.

KENNEDY, M.J., PRESTIGIACOMO, L.J., TYLOR, G., MAY, W.S. & DAVIDSON, N.E. (1992). Differential effects of bryostatial and phorbol ester on human breast cancer cell liner. *Cancer Res.*, **52**, 1278–1283.

KILEY, S., SCHAAP, D., PARKER, P., HSIEH, L. & JAKEN, S. (1990). Protein kinase C heterogeneity in $GH_4C_1$ rat pituitary cells. Characterization of a $Ca^2$-independent phorbol ester receptor. *J. Biol. Chem.*, **265**, 15704–15712.

KOBAYASHI, E., NAKANO, H., MORIMOTO, M. & TAMAOKI, T. (1989). Calphostin C (UCN-1028C), a novel microbial compound is a highly potent and specific inhibitor of protein kinase C. *Biochem. Biophys. Res. Commun.*, **159**, 548–553.

KOMADA, F., NISHIKAWA, M., UEMURA, Y., MORITA, K., HIDAKA, H. & SHIRAKAWA, S. (1991). Expression of three major protein kinase C isozymes in various types of human leukemic cells. *Cancer Res.*, **51**, 4271–4278.

KOPP, R., NOELKE, B., SAUTER, G., SHILDBERG, F.W., PAUMGARTNER, G. & PFEIFFER, A. (1991). Altered proitein kinase C activity in biopsies of human colonic adenomas and carcinomas. *Cancer Res.*, **51**, 205–210.

KRAFT, A.S., REEVES, J.A. & ASHENDEL, C.L. (1988). Differing modulation of protein kinase C by bryostatin 1 and phorbol esters in JB6 mouse epidermal cells. *J. Biol. Chem.*, **263**, 8437–8442.

KRAFT, A.S. & ANDERSON, W.B. (1983). Phorbol esters increase the amount of $Ca^{2+}$, phospholipid-dependent protein kinase associated with plasma membrane. *Nature*, **301**, 621–623.

KRAUSS, R.S., HOUSEY, G.M., JOHNSON, M.D. & WEINSTEIN, I.B. (1989). Disturbances in growth control and gene expression in a cell line that stably overproduces protein kinase C. *Oncogene*, **4**, 991–998.

KRAFT, A.S., SMITH, J.B. & BERKOW, R.L. (1986). Bryostatin, an activator of the calcium phospholipid-dependent protein kinase, blocks phorbol ester-induced differentiation of human promyelocytic leukaemia cells HL-60. *Proc. Natl Acad. Sci. USA*, **83**, 1334–1338.

LAUGHTON, C.A., DALE, I.L. & GESCHER, A. (1989*a*). Studies on bioactive compounds. 13. Synthesis and lack of growth-inhibitory properties of cyclohexane-1,2,4-triol 1,2-diesters, which resemble ring C of the phorbol ester molecule. *J. Med. Chem.*, **32**, 428–433.

LAUGHTON, C.A., BRADSHAW, T.D. & GESCHER, A. (1989*b*). Sterically hindered analogues of diacylglycerols. Synthesis, binding to the phorbol ester receptor and metabolism in A549 human lung carcinoma cells. *Int. J. Cancer*, **44**, 320–324.

LILLY, M., BROWN, C., PETTIT, G. & KRAFT, A. (1991). Bryostatin 1: potential anti-leukemic agent for chronic myelomonocytic leukemia. *Leukemia*, **5**, 283–287.

LINDNER, D., GSCHWENDT, M. & MARKS, F. (1991). Down-regulation of protein kinase C in Swiss 3T3 fibroblasts is independent of its phosphorylating activity. *Biochem. Biophys. Res. Commun.*, **176**, 1227–1231.

LOMIS, C.R. & BELL, R.M. (1988). Sangivamycin, a nucleoside analogue, is a potent inhibitor of protein kinase C. *J. Biol. Chem.*, **263**, 1682–1692.

MACKANOS, E.A., PETTIT, G.R. & RAMSDELL, J.S. (1991). Bryostatins selectivity regulate protein kinase C-mediated effects on $GH_4$ cell proliferation. *J. Biol. Chem.*, **266**, 11205–11212.

MAHONEY, C.W., AZZI, A. & HUANG, K.P. (1990). Effects of suramin, an anti-human immunodeficiency virus reverse transcriptase agent, on protein kinase C. *J. Biol. Chem.*, **265**, 5424–5428.

MARAIS, R.M. & PARKER, P.J. (1989). Purification and characterisation of bovine brain protein kinase C isotypes α, β and γ. *Eur. J. Biochem.*, **182**, 129–137.

MARX, M.H., PIANTADOSI, C., NOSEDA, A., DANIEL, L.W. & MODEST, E.J. (1988). Synthesis and evaluation of neoplastic cell growth inhibition of 1-N-alkylamide analogues of glycero-3-phosphocholine. *J. Med. Chem.*, **31**, 858–863.

MAY, W.S., SHARKIS, S.J., ESA, A.H., GEBBIA, V., KRAFT, A.S., PETTIT, G.R. & SENSEBRENNER, L.L. (1987). Antineoplastic bryostatins are multipotential stimulators of human hematopoietic progenitor cells. *Proc. Natl Acad. Sci. USA*, **84**, 8483–8487.

MCBAIN, J.,A., PETTIT, G.R. & MUELLER, G.C. (1988). Bryostatin 1 antagonizes the terminal differentiating action of 12-*O*-tetradecanoylphorbol-13-acetate in a human colon cancer cell. *Carcinogenesis*, **9**, 123–129.

MCSWINE-KENNICK, R.L., MCKEEGAN, E.M., JOHNSON, M.D. & MORIN, M.J. (1991). Phorbol diester-induced alterations in the expression of protein kinase C isozymes and their mRNAs. *J. Biol. Chem.*, **266**, 15135–15143.

MEGEDISH, T. & MAZUREK, N. (1989). A mutant protein kinase C that can transform fibroblasts. *Nature*, **342**, 807–811.

MERRILL, A.H., NUMKAR, S., MENALDINO, D., HANNUN, Y.A., LOOMIS, C., BELL, R.M., TYAGI, S.R., LAMBETH, J.D., STEVENS, V.L., HUNTER, R. & LIOTTA, D.C. (1989). Structural requirements for long-chain (sphingoid) base inhibition of protein kinase C *in vitro* and for the cellular effects of these compounds. *Biochemistry*, **28**, 3138–3145.

MEYER, T., REGENASS, U., FABBRO, D., ALTERI, E., RÖSEL, J., MÜLLER, M., CARAVATTI, G. & MATER, A. (1989). A derivative of staurosporine (CGP 41251) shows selectivity for protein kinase C inhibition and *in vitro* anti-proliferative as well as *in vivo* anti-tumour activity. *Int. J. Cancer*, **43**, 851–856.

MOCHLY-ROSEN, D., KHANER, H., LOPEZ, J. & SMITH, B.L. (1991). Intracellular receptors for activated protein kinase C. *J. Biol. Chem.*, **266**, 14866–14868.

MOLLEYRES, L.P. & RANDO, R.R. (1988). Structural studies on the diglyceride-mediated activation of protein kinase C. *J. Biol. Chem.*, **263**, 14832–14838.

MORI, T., TAKAI, Y., MINAKUCHI, R., YU, B. & NISHIZUKA, Y. (1983). Inhibitory action of chloropromazine, dibucaine and other phospholipid-interacting drugs on protein kinase C. *J. Biol. Chem.*, **255**, 8378–8380.

MORIN, M.J., KREUTTER, D., RASMUSSEN, H. & SARTORELLI, A.C. (1987). Disparate effects of activators of protein kinase C on HL-60 promyelocytic leukemia cell differentiation. *J. Biol. Chem.*, **262**, 11758–11763.

NISHIKAWA, M., KOMADA, F., UEMURA, Y., HIDAKA, H. & SHIRAKAWA, S. (1990). Decreased expression of type II protein kinase C in HL-60 variant cells resistant to induction of cell differentiation by phorbol diester. *Cancer Res.*, **50**, 621–626.

NISHIZUKA, Y. (1988). The molecular heterogeneity of protein kinase C and its implications for cellular regulation. *Nature*, **334**, 661–665.

NISHIZUKA, Y. (1989). The protein kinase C family: heterogeneity and its implications. *Annu. Rev. Biochem.*, **58**, 31–44.

OBEID, L.M., OKAZAKI, T., KAROLAK, L.A. & HANNUN, U.A. (1990). Transcriptional regulation of protein kinase C by 1,25-dihydroxyvitamin $D_3$ in HL-60 cells. *J. Biol. Chem.*, **265**, 2370–2374.

O'BRIAN, C.A., LISKAMP, R.M., SOLOMON, D.H. & WEINSTEIN, I.B. (1985). Inhibition of protein kinase C by tamoxifen. *Cancer Res.*, **45**, 2462–2465.

O'BRIAN, C.A., VOGEL, V.G., SINGLETARY, S.E. & WARD, N.E. (1989). Elevated protein kinase C expression in human breast tumor biopsies relative to normal breast tissue. *Cancer Res.*, **49**, 3215–3217.

OKOSHI, H., HAKOMORI, S., NISAR, M., ZHOU, Q., KIMURI, S., TASHIRO, K. & IGARASHI, Y. (1991). Cell membrane signaling as target in cancer therapy II: inhibitory effect of N,N,N-trimethylsphingosine on metastatic potential of murine B16 melanoma cell line through blocking of tumor cell-dependent platelet aggregation. *Cancer Res.*, **51**, 6019–6024.

ONO, Y., FUJII, T., OGITA, K., KIKKAWA, U., IGARISHI, K. & NISHIZUKA, Y. (1989). Protein kinase C-ξ subspecies from rat brain: its structure, expression and properties. *Proc. Natl Acad. Sci. USA*, **86**, 3099–3103.

PARKER, P.J., KOUR, G., MARAIS, R.M., MITCHELL, F., PEARS, C.J., SCHAAP, D., STABEL, S. & WEBSTER, C. (1989). Protein kinase C – a family affair. *Mol. Cell. Endocrinol.*, **65**, 1–11.

PASTI, G., RIVEDAL, E., YUSPA, S.H., HERALD, C.L., PETTIT, G.R. & BLUMBERG, P.M. (1988). Contrasting duration of inhibition of cell-cell communication in primary mouse epidermal cells by phorbol 12,13-dibutyrate and by bryostatin 1. *Cancer Res.*, **48**, 447–451.

PEARS, C. & PARKER, P.J. (1991). Down-regulation of a kinase-defective PKC-α. *FEBS Lett.*, **284**, 120–122.

PEARSON, J.D., DEWALD, D.B., MATTHEWS, W.R., MOZIER, N.M., ZÜRCHER-NEELY, H.A., HEINRIKSON, R.L., MORRIS, M.A., MCCUBBIN, W.D., MCDONALD, J.R., FRASER, E.D., VOGEL, H.J., KAY, C.M. & WALSH, M.P. (1990). Amino acid sequence and characterization of a protein inhibitor of protein kinase C. *J. Biol. Chem.*, **265**, 4585–4591.

PELOSIN, J.M., KERAMIDAS, M., SOUVIGNET, C. & CHAMBAZ, E.M. (1990). Differential inhibition of protein kinase C subtypes. *Biochem. Biophys. Res. Comun.*, **169**, 1040–1048.

PERSONS, D.A., WILKISIN, W.O., BELL, R.M. & FINN, O.J. (1988). Altered growth regulation and enhanced tumorigenicity of NIH3T3 fibroblasts transfected with protein kinase C-I c-DNA. *Cell*, **52**, 447–458.

PETTIT, G.R. (1991). The Bryostatins. In *Progress in the Chemistry of Organic Natural Products*. Vol. 57. Herz, W., Kirby, G.W., Steglich, W. & Tamm, C. (eds), Springer-Verlag: Wien, New York, pp. 154–195.

PETTIT, G.R., DAY, J.F., HARTWELL, J.L. & WOOD, H.B. (1970). Antineoplastic components of marine animals. *Nature*, **227**, 962–963.

PETTIT, G.R., HERALD, C.L., DOUBEK, D.L., HERALD, D.L., ARNOLD, E. & CLARDY, J. (1982). Isolation and structure of bryostatin 1. *J. Am. Chem. Soc.*, **104**, 6846–6848.

PONTREMOLI, S., MELLONI, E., SPARATORE, B., MICHETTI, M., SALAMINO, F. & HORECKER, B.L. (1990). Isozymes of protein kinase C in human neutrophils and their modification by two endogenous proteinases. *J. Biol. Chem.*, **265**, 706–712.

POSADA, J.A., MCKEEGAN, E.M., WORTHINGTON, K.F., MORIN, M.J., JAKEN, S. & TRITTON, T.R. (1989). Human multidrug resistant KB cells overexpress protein kinase C: involvement in drug resistance. *Cancer Commun.*, **1**, 285–292.

POWIS, G. (1991). Signalling targets for anticancer drug development. *Trends Pharmacol. Sci.*, **12**, 188–194.

RANDO, R.R. & KISHI, Y. (1992). Structural basis of protein kinase C activation by diacylglycerols and tumor promoters. *Biochem.*, **31**, 2211–2218.

REDDY, A.L. & FIALKOW, P.J. (1990). Evidence that weak promotion of carcinogen-initiated cells prevents their progression of malignancy. *Carcinogenesis*, **11**, 2123–2126.

RODRIGUEZ-PENA, A. & ROZENGURT, E. (1984). Disappearance of $Ca^{2+}$-sensitive, phospholipid-dependent protein kinase activity in phorbol ester-treated 3T3 cells. *Biochem. Biophys. Res. Commun.*, **120**, 1053–1059.

ROTENBERG, S.A., SMILEY, S., UEFFING, M., KRAUSS, R.S., CHEN, L.B. & WEINSTEIN, I.B. (1990). Inhibition of rodent protein kinase C by the anticarcinoma agent dequalinium. *Cancer Res.*, **50**, 677–685.

RYVES, W.J., EVANS, A.T., OLIVIER, A.R., PARKER, P.J. & EVANS, F.J. (1991). Activation of the PKC-isotypes α, $\beta_1$, γ, δ and ε by phorbol esters of different biological activities. *FEBS Lett.*, **288**, 5–9.

SAKANOUE, Y., HATADA, T., KUSUNOKI, M., YANAGI, H., YAMAMURA, T. & UTSUNOMIYA, J. (1991). Protein kinase C activity as marker for colorectal cancer. *Int. J. Cancer*, **48**, 803–806.

SAKO, T., YUSPA, S.H., HERALD, C.L., PETTIT, G.R. & BLUMBERG, P.M. (1987). Partial parallelism and partial blockade by bryostatin 1 of effects of phorbol ester tumor promoters on primary mouse epidermal cells. *Cancer Res.*, **47**, 5445–5450.

SCHAAP, D. & PARKER, P.J. (1990). Expression, purification, and characterization of protein kinase C-ε. *J. Biol. Chem.*, **265**, 7301–7307.

SCHAAP, D., HSUAN, J., TOTTY, N. & PARKER, P.J. (1990). Proteolytic activation of protein kinase C-ε. *Eur. J. Biochem.*, **191**, 431–435.

SCHAAP, D., PARKER, P.J., BRISTOL, A., KRIZ, R. & KNOPF, J. (1989). Unique substrate specificity and regulatory properties of protein kinaser C-ε: a rationale for diversity. *FEBS Lett.*, **243**, 351–357.

SCHUCHTER, L.M., ESA, A.H.,, STRATFORD MAY, W., LAULIS, M.K., PETTIT, G.R. & HESS, A.D. (1991). Successful treatment of murine melanoma with bryostatin 1. *Cancer Res.*, **51**, 682–687.

SCHWARTZ, G.K., REDWOOD, S.M., OHNUMA, T., HOLLAND, J.F., DROLLER, M.J. & LIU, B.C.S. (1990). Inhibition of invasion of invasive human bladder carcinoma cells by protein kinase C inhibitor stuarosporine. *J. Natl Cancer Inst.*, **82**, 1753–1756.

SEKIGUCHI, K., TSUKUDA, M., OGITA, K., KIKKAWA, U. & NISHIZUKA, Y. (1987). Three distinct forms of rat brain protein kinase C: differential response to unsaturated fatty acids. *Biochem. Biophys. Res. Commun.*, **145**, 797–802.

SHIMIZU, N., OHTA, M., FUJIWARA, C., SGARA, J., MOCHIZUKI, N., ODA, T. & UTIYAMA, H. (1991). Expression of a novel immediate early gene during 12-*O*-tetradecanoylphorbol-13-acetate-induced macrophagic differentiation of HL-60 cells. *J. Biol. Chem.*, **266**, 12157–12161.

SHIMIZU, Y. & SHIMIZU, N. (1989). Cell genetic evidence of correlation of intracellular translocation of protein kinase C (PKC) and PKC-mediated phosphorylation of 80-kDa protein with mitogenic action of tumor promoters. *Somat. Cell Mol. Genet.*, **15**, 321–329.

SMITH, J.A., SMITH, L. & PETTIT, G.R. (1985). Bryostatins: potent new mitogens that mimic phorbol ester tumor promoters. *Biochem. Biophys. Res. Commun.*, **131**, 939–945.

STABEL, S. & PARKER, P.J. (1991). Protein kinase C. *Pharmac. Ther.*, **51**, 71–95.

STEVENS, V.L., NIMKAR, S., JAMISON, W.C.L., LIOTTA, D.C. & MERRILL, A.H. (1990). Characteristics of the growth inhibition and cytotoxicity of long-chain (sphingoid) bases for chinese hamster ovary cells: evidence for an involvement of protein kinase C. *Biochim. Biophys. Acta*, **1051**, 37–45.

STRULOVICI, B., DANIEL-ISSAKANI, S. & BAXTER, G., KNOPF, J., SULTZMAN, L., CHERWINSKI, H., NESLOV, J., WEBB, D.R. & RANSOM, J. (1991). Distinct mechanisms of regulation of protein kinase Cε by hormones and phorbol diesters. *J. Biol. Chem.*, **266**, 168–173.

SULLIVAN, J.P., CONNOR, J.R., SHEARER, B.G. & BURCH, R.M. (1991). 2,6-Diamino-N-([1-oxotridecyl)-2-piperidinyl]methyl) hexanamide (NPC15437): a selective inhibitor of protein kinase C. *Agents and Actions*, **34**, 142–144.

TAMAOKI, T. & NAKANO, H. (1990). Potent and specific inhibitors of protein kinase C of microbial origin. *Biotechnology*, **8**, 732–735.

TAMAOKI, T., NOMOTO, H., TAKAHASHI, I., KATO, Y., MORIMOTO, M. & TOMITA, F. (1986). Staurosporine, a potent inhibitor of protein kinase C from strepotomyces. *Biochem. Biophys. Res. Commun .*, **135**, 397–402.

TRILIVAS, I., MCDONOUGH, P.M. & BROWN, J.H. (1991). Dissociation of protein kinase C redistribution from the phosphorylation of its subtrates. *J. Biol. Chem.*, **266**, 8431–8438.

TRITTON, T.R. & HICKMAN, J.A. (1990). How to kill cancer cells: membranes and cell signalling as targets in cancer chemotherapy. *Cancer Cells*, **2**, 95–105.

ÜBERALL, F., OBERHUBER, H., MALY, K., ZAKNUN, J., DEMUTH, L. & GRUNICKE, H.H. (1991*a*). Hexadecylphosphocholine inhibits inositol phosphate formation and protein kinase C activity. *Cancer Res.*, **51**, 807–812.

ÜBERALL, F., MALY, K., EGLE, A., DOPPLER, W., HOFMANN, J. & GRUNICKE, H.H. (1991*b*). Inhibition of cell proliferation, protein kinase C, and phorbol ester-induced *fos* expression by the dihydropyridine derivative B859-35. *Cancer Res.*, **51**, 5821–5825.

VALETTE, A., GAS, N., JOZAN, S., ROUBINET, F., DUPONT, M.A. & BAYARD, F. (1987). Influence of 12-*O*-tetradecanoylphorbol-13-acetate on proliferation and maturation of human breast carcinoma cells (MCF-7): relationship to cell cycle events. *Cancer Res.*, **47**, 1615–1620.

VANDENBARK, G.R. & NIEDEL, J.E. (1984). Phorbol diesters and cellular differentiation. *J. Natl Cancer Inst.*, **73**, 1013–1019.

WARREN, B.S., KAMANO, Y., PETTIT, G.R. & BLUMBERG, P.M. (1988). Mimicry of bryostatin 1 induced phosphorylation patterns in HL-60 cells by high phorbol ester concentrations. *Cancer Res.*, **48**, 5984–5988.

WEINSTEIN, I.B. (1988). The origins of human cancer: molecular mechanisms of carcinogenesis and their implications for cancer prevention and treatment – Twenty-seventh G.H.A. Clowes Memorial Award Lecture. *Cancer Res.*, **48**, 4135–4143.

WENDER, P.A., CRIBBS, C.M., KOEHLER, K.F., SHARKEY, N.A., HERALD, C.L., KAMANO, Y., PETTIT, G.R. & BLUMBERG, P.M. (1988). Modeling of the bryostatins to the phorbol ester pharmacophore on protein kinase C. *Proc. Natl Acad. Sci. USA*, **85**, 7197–7201.

WISE, B.C. & KUO, J.F. (1983). Modes of inhibition by acylcarnitines, adriamycin and trifluoperazine of cardiac phospholipid-sensitive calcium-dependent protein kinase. *Biochem. Pharmacol.*, **32**, 1259–1265.

WOLFMAN, A. & MAKARA, I.G. (1987). Elevated levels of diacylglycerol and decreased phorbol ester sensitivity in *ras*-transformed fibroblasts. *Nature*, **325**, 359–361.

YAMANISHI, D.T., GRAHAM, M., BUCKMEIER, J.A. & MEYSKENS, F.L. (1991). The differential expression of protein kinase C genes in normal human neonatal melanocytes and metastatic melanomas. *Carcinogenesis*, **12**, 105–109.

YOUNG, S., PARKER, P.J., ULLRICH, A. & STABEL, S. (1987). Downregulation of protein kinase C is due to an increased rate of degradation. *Biochem. J.*, **244**, 775–779.

YOSHIZAWA, S., FUJIKI, H., SUGURI, H., SUGANAMA, M., NAKASAYU, M., MATSUSHIMA, R. & SUGIMURA. T. (1990). Tumor-promoting activity of staurosporine, a protein kinase inhibitor, on mouse skin. *Cancer Res.*, **50**, 4974–4978.

ZHENG, B., OISHI, K., SHOJI, M., EIBL, H., BERDEL, W.E., HAJDU, J., VOGLER, W.R. & KUO, J.F. (1990). Inhibition of protein kinase C, (sodium plus potassium)-activated adenosine triophosphatase and sodium pump by synthetic phospholipid analogues. *Cancer Res.*, **50**, 3025–3031.


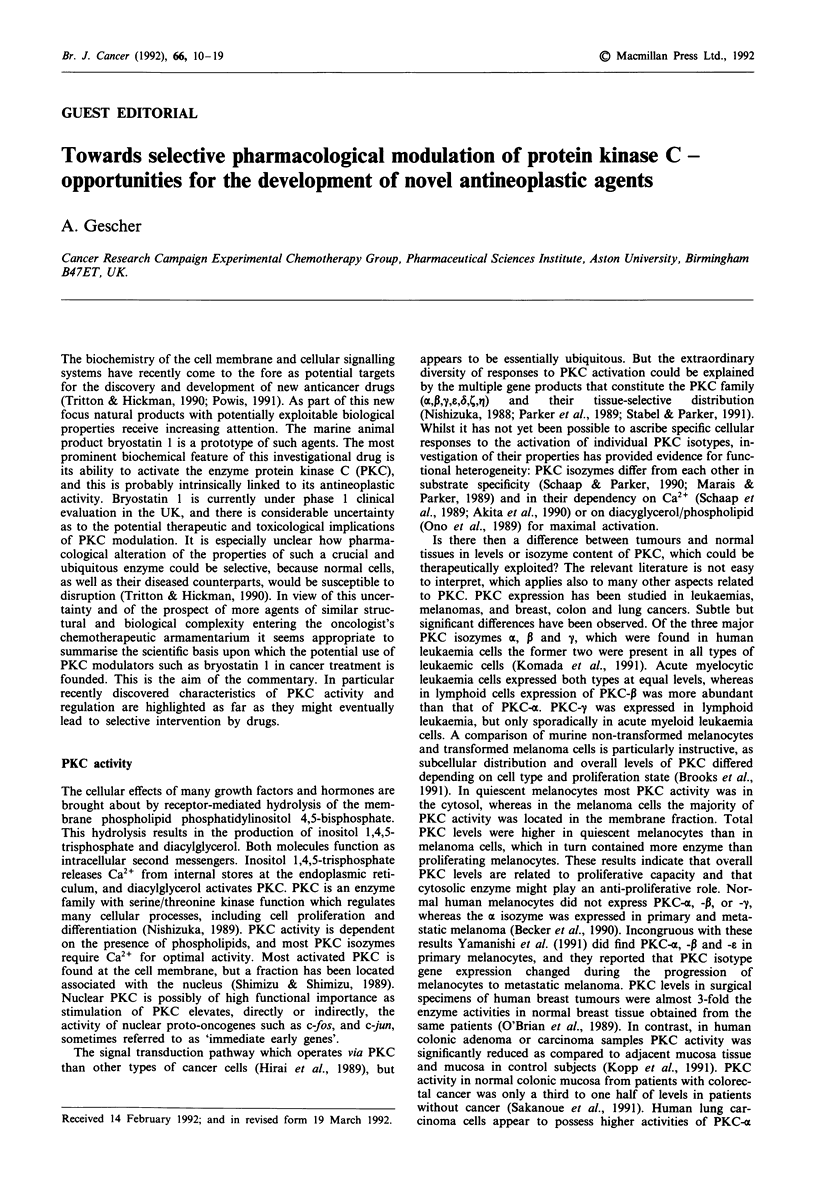

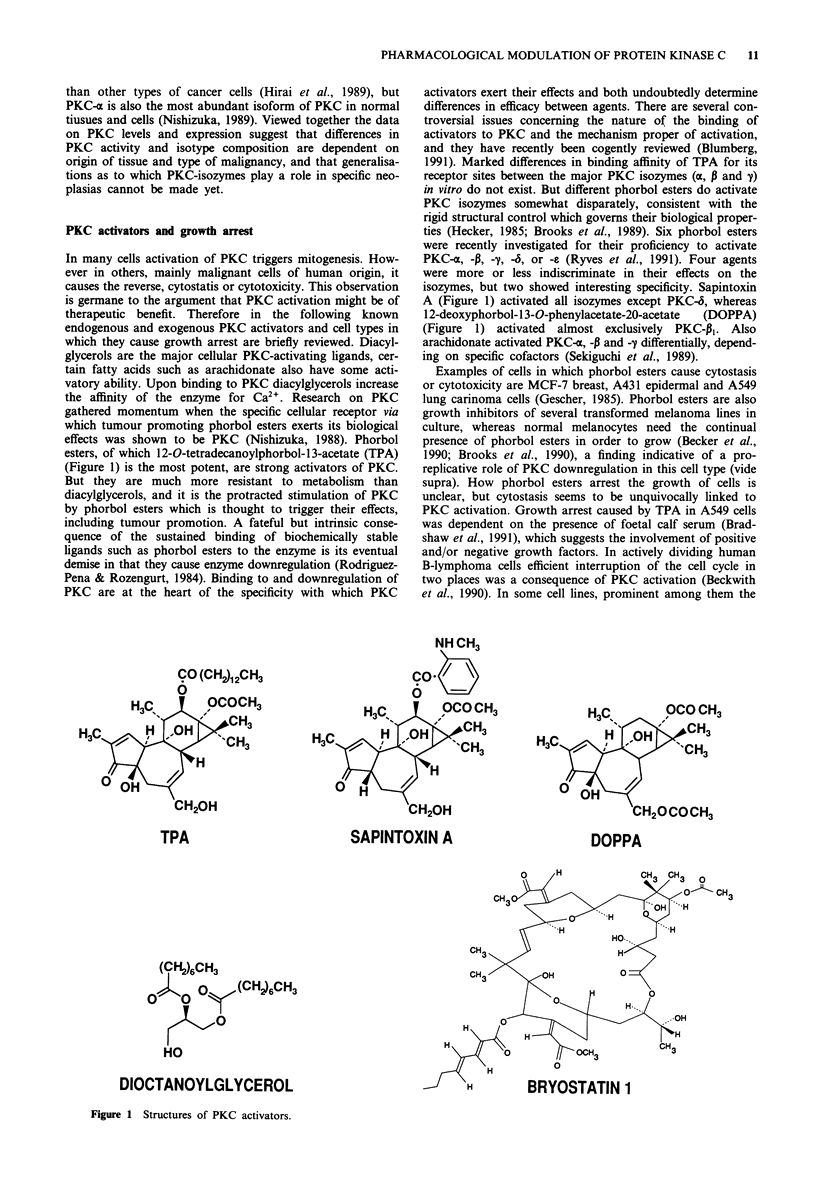

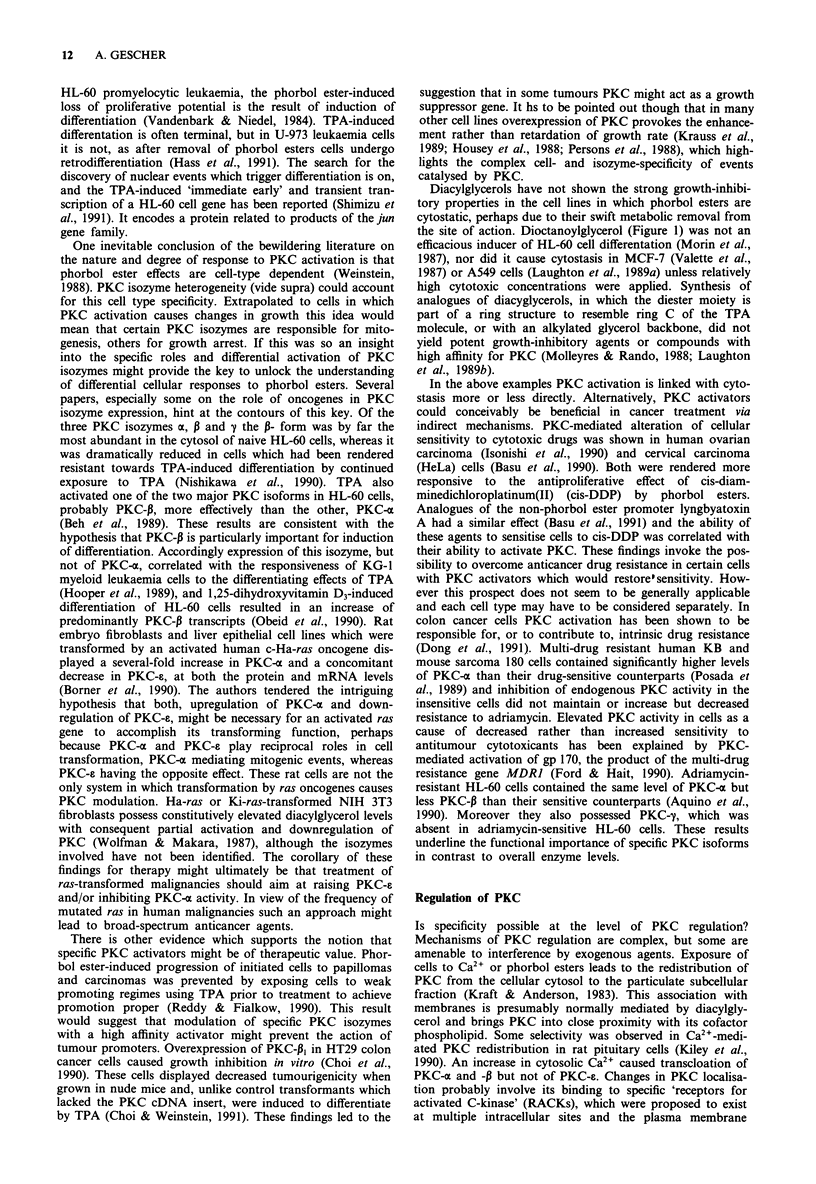

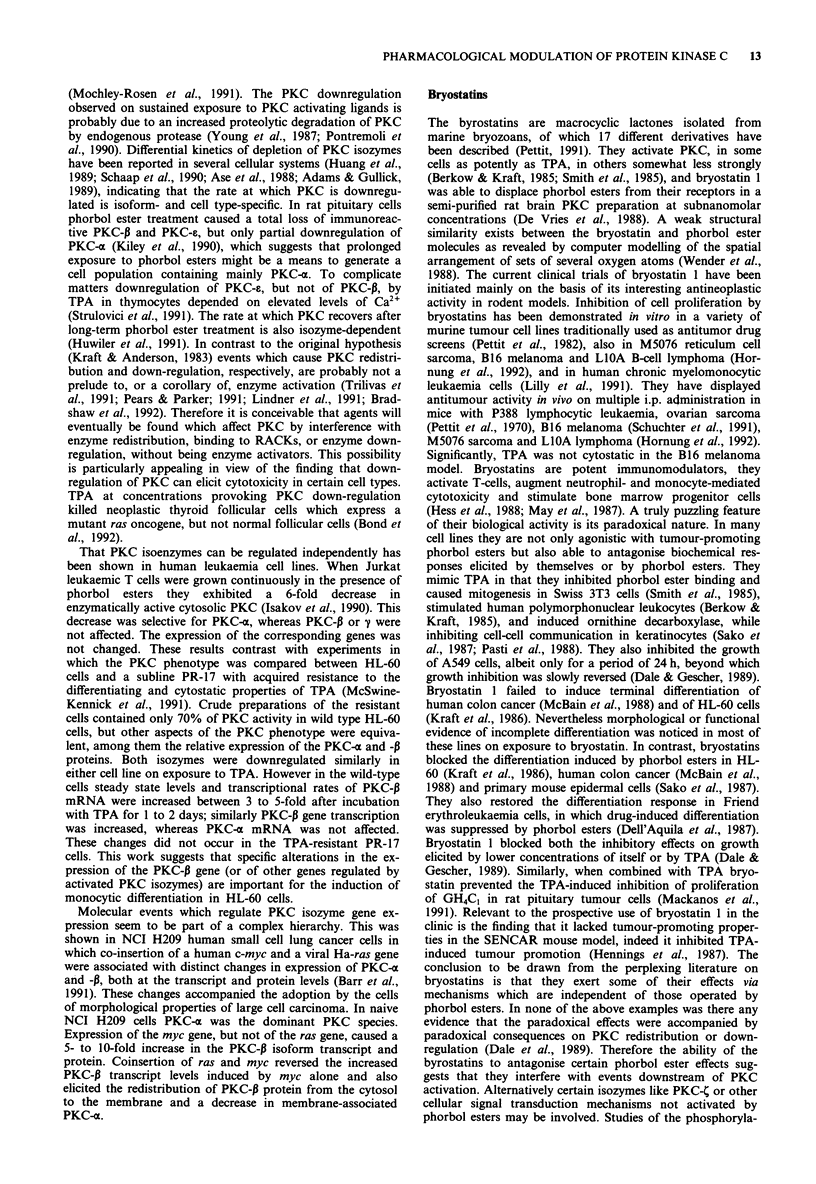

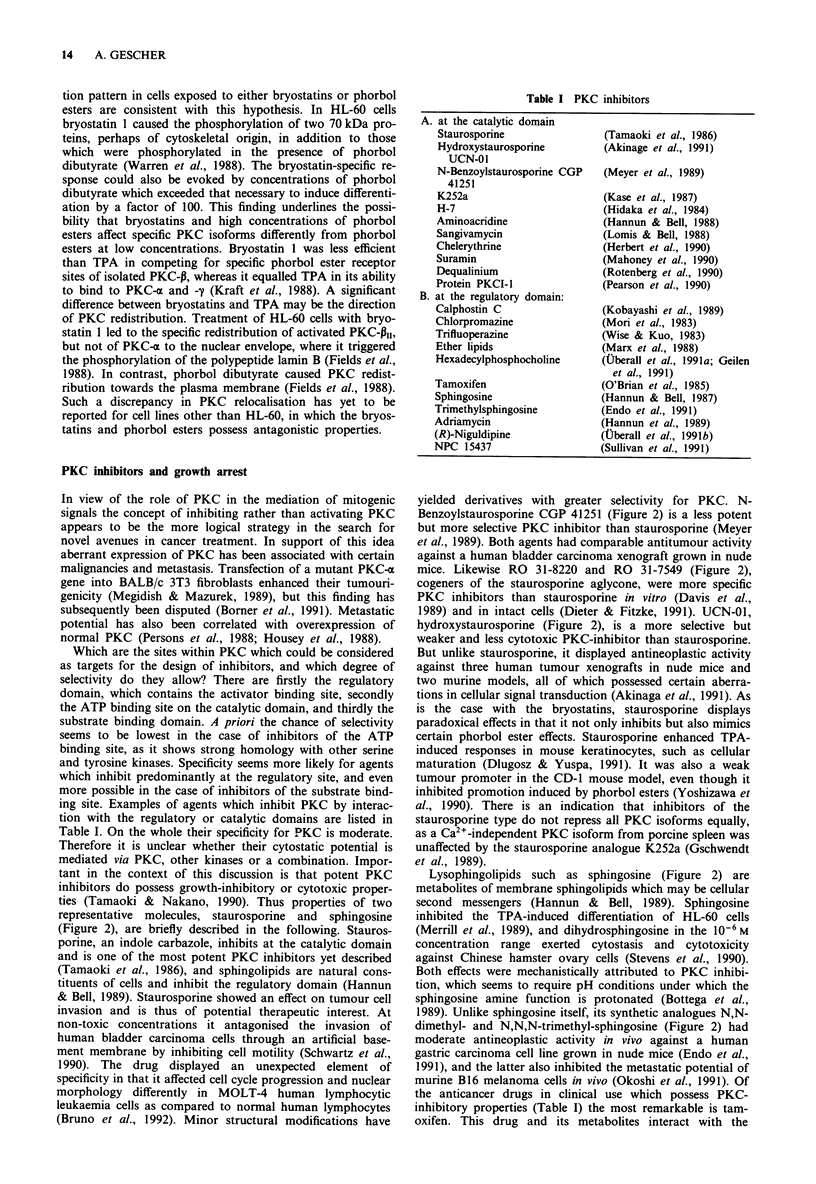

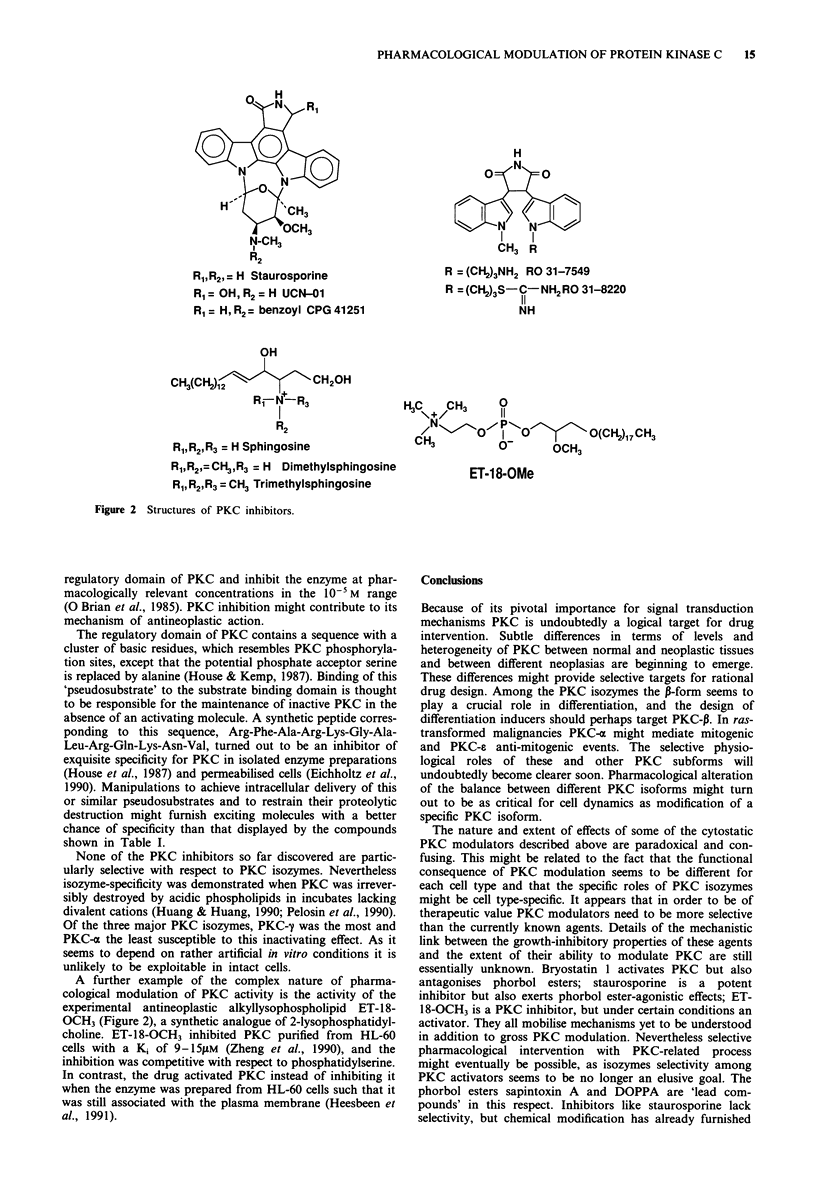

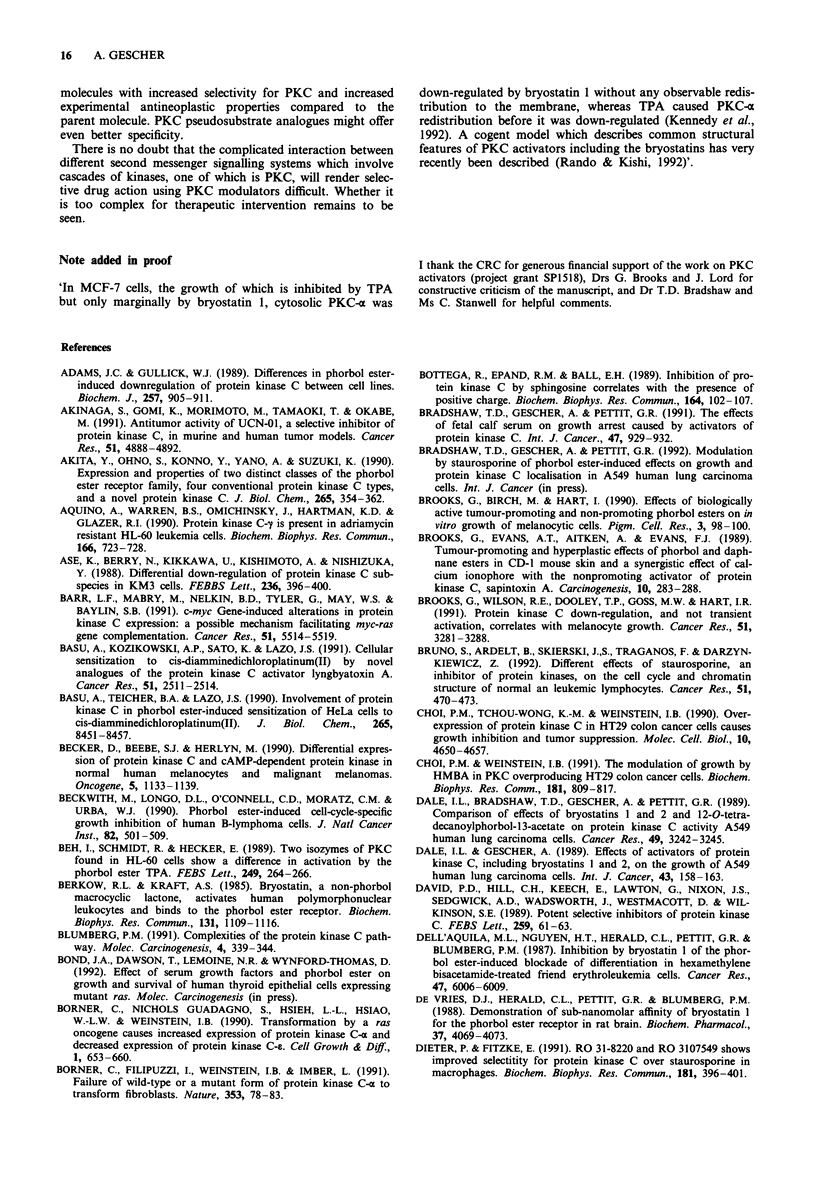

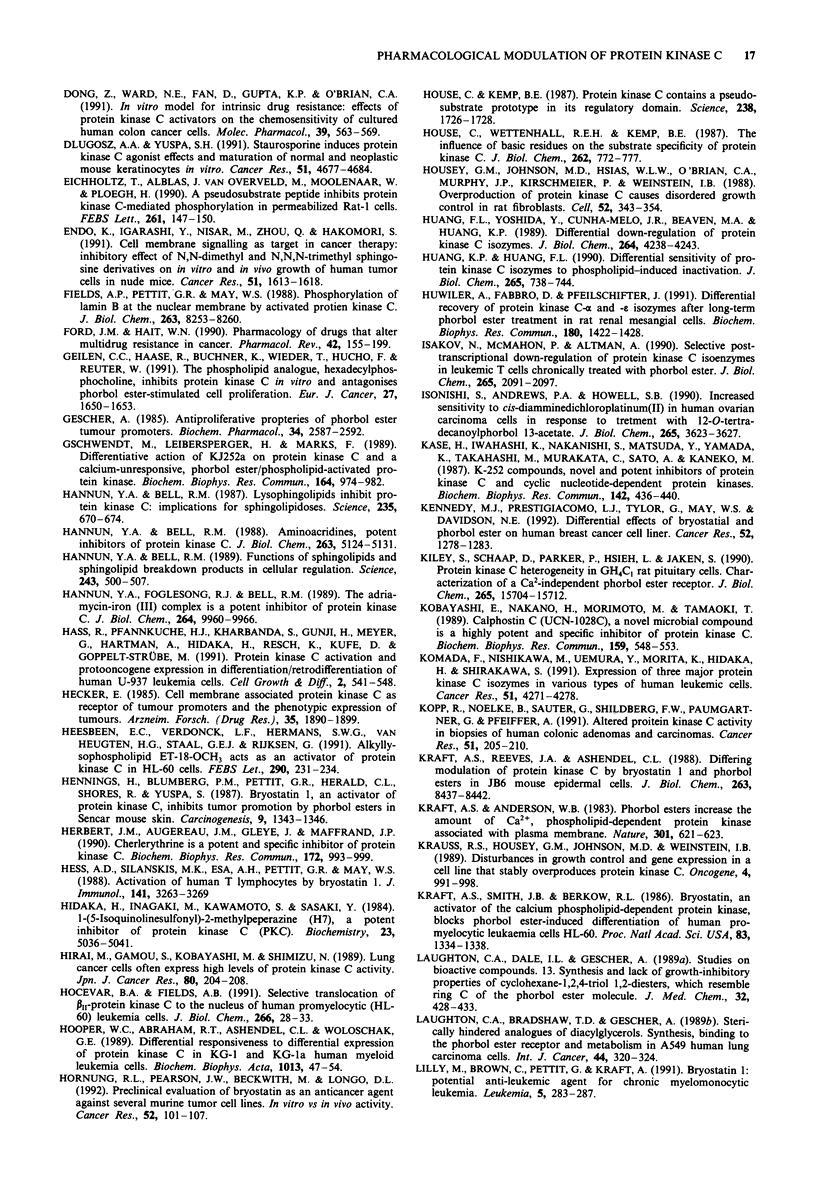

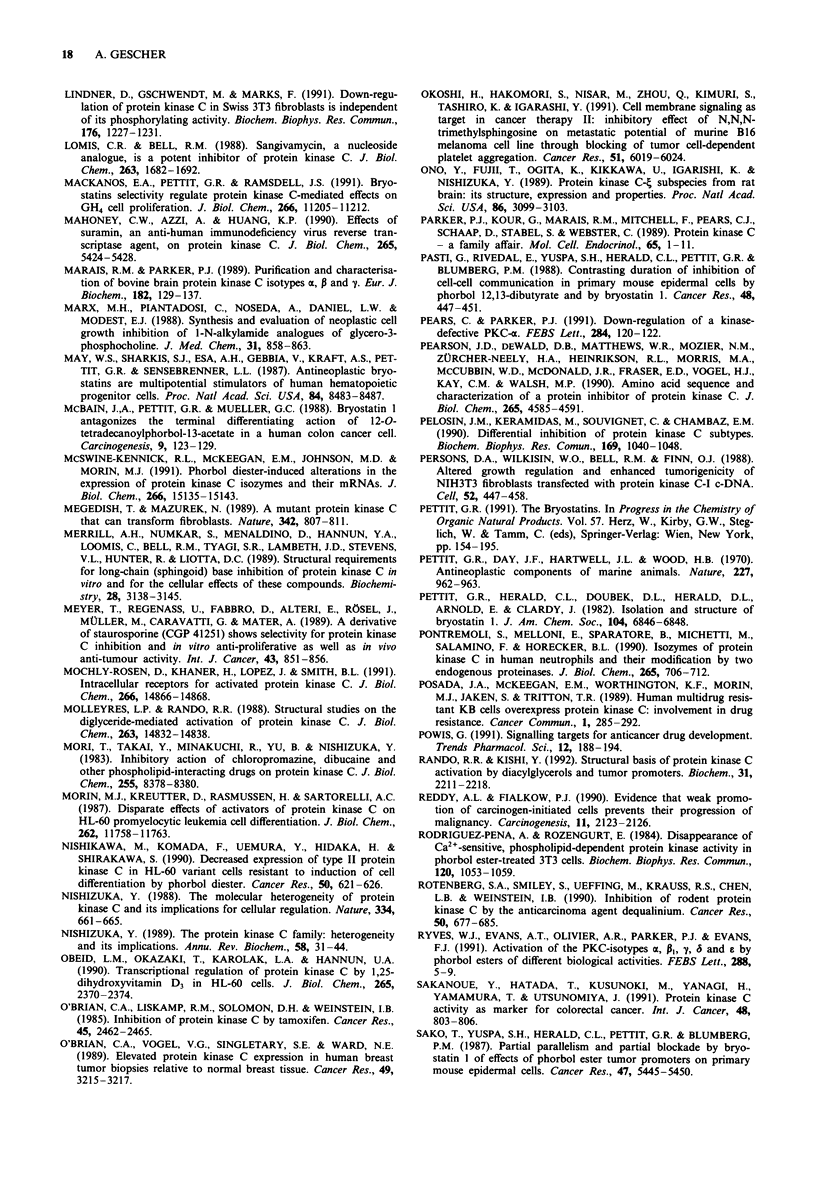

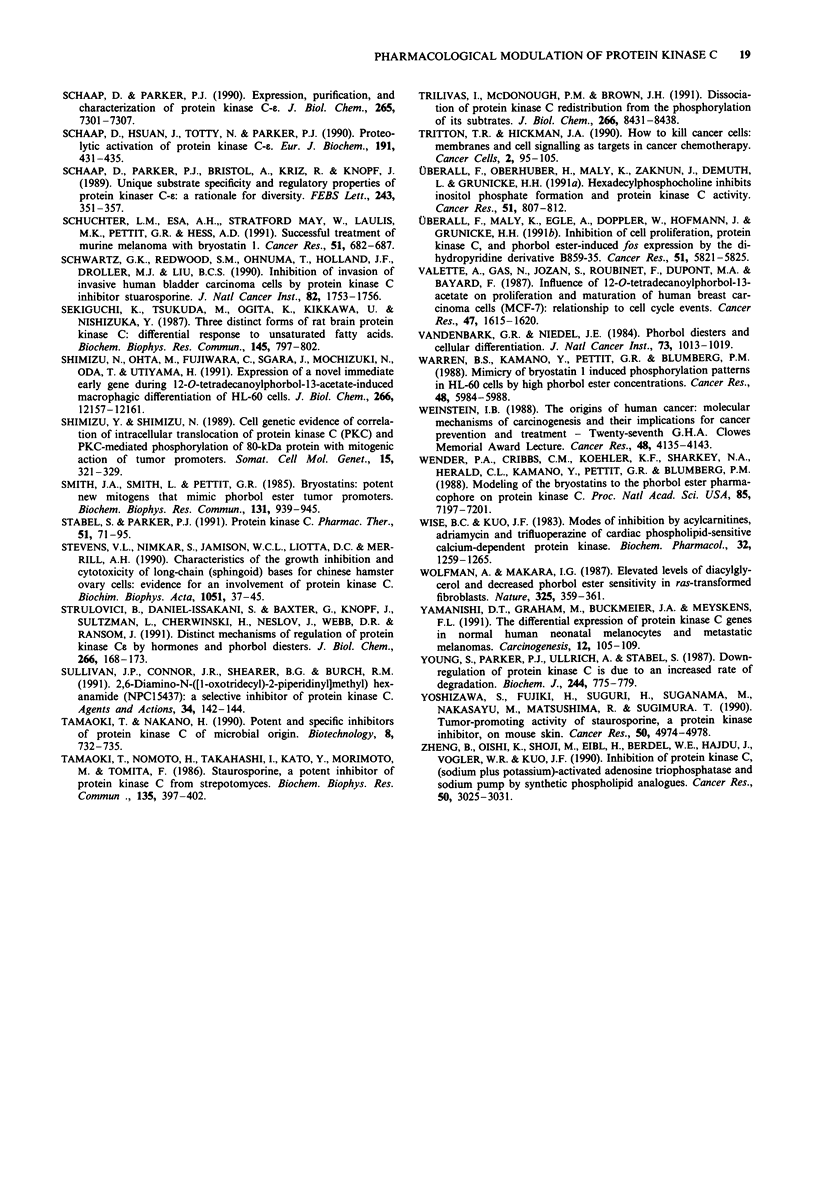


## References

[OCR_00926] Adams J. C., Gullick W. J. (1989). Differences in phorbol-ester-induced down-regulation of protein kinase C between cell lines.. Biochem J.

[OCR_00931] Akinaga S., Gomi K., Morimoto M., Tamaoki T., Okabe M. (1991). Antitumor activity of UCN-01, a selective inhibitor of protein kinase C, in murine and human tumor models.. Cancer Res.

[OCR_00937] Akita Y., Ohno S., Konno Y., Yano A., Suzuki K. (1990). Expression and properties of two distinct classes of the phorbol ester receptor family, four conventional protein kinase C types, and a novel protein kinase C.. J Biol Chem.

[OCR_00943] Aquino A., Warren B. S., Omichinski J., Hartman K. D., Glazer R. I. (1990). Protein kinase C-gamma is present in adriamycin resistant HL-60 leukemia cells.. Biochem Biophys Res Commun.

[OCR_00949] Ase K., Berry N., Kikkawa U., Kishimoto A., Nishizuka Y. (1988). Differential down-regulation of protein kinase C subspecies in KM3 cells.. FEBS Lett.

[OCR_00954] Barr L. F., Mabry M., Nelkin B. D., Tyler G., May W. S., Baylin S. B. (1991). c-myc gene-induced alterations in protein kinase C expression: a possible mechanism facilitating myc-ras gene complementation.. Cancer Res.

[OCR_00960] Basu A., Kozikowski A. P., Sato K., Lazo J. S. (1991). Cellular sensitization to cis-diamminedichloroplatinum(II) by novel analogues of the protein kinase C activator lyngbyatoxin A.. Cancer Res.

[OCR_00966] Basu A., Teicher B. A., Lazo J. S. (1990). Involvement of protein kinase C in phorbol ester-induced sensitization of HeLa cells to cis-diamminedichloroplatinum(II).. J Biol Chem.

[OCR_00972] Becker D., Beebe S. J., Herlyn M. (1990). Differential expression of protein kinase C and cAMP-dependent protein kinase in normal human melanocytes and malignant melanomas.. Oncogene.

[OCR_00978] Beckwith M., Longo D. L., O'Connell C. D., Moratz C. M., Urba W. J. (1990). Phorbol ester-induced, cell-cycle-specific, growth inhibition of human B-lymphoma cell lines.. J Natl Cancer Inst.

[OCR_00984] Beh I., Schmidt R., Hecker E. (1989). Two isozymes of PKC found in HL-60 cells show a difference in activation by the phorbol ester TPA.. FEBS Lett.

[OCR_00989] Berkow R. L., Kraft A. S. (1985). Bryostatin, a non-phorbol macrocyclic lactone, activates intact human polymorphonuclear leukocytes and binds to the phorbol ester receptor.. Biochem Biophys Res Commun.

[OCR_00995] Blumberg P. M. (1991). Complexities of the protein kinase C pathway.. Mol Carcinog.

[OCR_01012] Borner C., Filipuzzi I., Weinstein I. B., Imber R. (1991). Failure of wild-type or a mutant form of protein kinase C-alpha to transform fibroblasts.. Nature.

[OCR_01005] Borner C., Guadagno S. N., Hsieh L. L., Hsiao W. L., Weinstein I. B. (1990). Transformation by a ras oncogene causes increased expression of protein kinase C-alpha and decreased expression of protein kinase C-epsilon.. Cell Growth Differ.

[OCR_01017] Bottega R., Epand R. M., Ball E. H. (1989). Inhibition of protein kinase C by sphingosine correlates with the presence of positive charge.. Biochem Biophys Res Commun.

[OCR_01021] Bradshaw T. D., Gescher A., Pettit G. R. (1991). The effect of fetal calf serum on growth arrest caused by activators of protein kinase C.. Int J Cancer.

[OCR_01032] Brooks G., Birch M., Hart I. R. (1990). Effects of biologically active tumour-promoting and non-promoting phorbol esters on in vitro growth of melanocytic cells.. Pigment Cell Res.

[OCR_01036] Brooks G., Evans A. T., Aitken A., Evans F. J. (1989). Tumour-promoting and hyperplastic effects of phorbol and daphnane esters in CD-1 mouse skin and a synergistic effect of calcium ionophore with the non-promoting activator of protein kinase C, sapintoxin A.. Carcinogenesis.

[OCR_01043] Brooks G., Wilson R. E., Dooley T. P., Goss M. W., Hart I. R. (1991). Protein kinase C down-regulation, and not transient activation, correlates with melanocyte growth.. Cancer Res.

[OCR_01056] Choi P. M., Tchou-Wong K. M., Weinstein I. B. (1990). Overexpression of protein kinase C in HT29 colon cancer cells causes growth inhibition and tumor suppression.. Mol Cell Biol.

[OCR_01062] Choi P. M., Weinstein I. B. (1991). The modulation of growth by HMBA in PKC overproducing HT29 colon cancer cells.. Biochem Biophys Res Commun.

[OCR_01067] Dale I. L., Bradshaw T. D., Gescher A., Pettit G. R. (1989). Comparison of effects of bryostatins 1 and 2 and 12-O-tetradecanoylphorbol-13-acetate on protein kinase C activity in A549 human lung carcinoma cells.. Cancer Res.

[OCR_01073] Dale I. L., Gescher A. (1989). Effects of activators of protein kinase C, including bryostatins 1 and 2, on the growth of A549 human lung carcinoma cells.. Int J Cancer.

[OCR_01081] Davis P. D., Hill C. H., Keech E., Lawton G., Nixon J. S., Sedgwick A. D., Wadsworth J., Westmacott D., Wilkinson S. E. (1989). Potent selective inhibitors of protein kinase C.. FEBS Lett.

[OCR_01086] Dell'Aquila M. L., Nguyen H. T., Herald C. L., Pettit G. R., Blumberg P. M. (1987). Inhibition by bryostatin 1 of the phorbol ester-induced blockage of differentiation in hexamethylene bisacetamide-treated Friend erythroleukemia cells.. Cancer Res.

[OCR_01097] Dieter P., Fitzke E. (1991). RO 31-8220 and RO 31-7549 show improved selectivity for protein kinase C over staurosporine in macrophages.. Biochem Biophys Res Commun.

[OCR_01110] Dlugosz A. A., Yuspa S. H. (1991). Staurosporine induces protein kinase C agonist effects and maturation of normal and neoplastic mouse keratinocytes in vitro.. Cancer Res.

[OCR_01104] Dong Z. Y., Ward N. E., Fan D., Gupta K. P., O'Brian C. A. (1991). In vitro model for intrinsic drug resistance: effects of protein kinase C activators on the chemosensitivity of cultured human colon cancer cells.. Mol Pharmacol.

[OCR_01115] Eichholtz T., Alblas J., van Overveld M., Moolenaar W., Ploegh H. (1990). A pseudosubstrate peptide inhibits protein kinase C-mediated phosphorylation in permeabilized Rat-1 cells.. FEBS Lett.

[OCR_01121] Endo K., Igarashi Y., Nisar M., Zhou Q. H., Hakomori S. (1991). Cell membrane signaling as target in cancer therapy: inhibitory effect of N,N-dimethyl and N,N,N-trimethyl sphingosine derivatives on in vitro and in vivo growth of human tumor cells in nude mice.. Cancer Res.

[OCR_01128] Fields A. P., Pettit G. R., May W. S. (1988). Phosphorylation of lamin B at the nuclear membrane by activated protein kinase C.. J Biol Chem.

[OCR_01133] Ford J. M., Hait W. N. (1990). Pharmacology of drugs that alter multidrug resistance in cancer.. Pharmacol Rev.

[OCR_01137] Geilen C. C., Haase R., Buchner K., Wieder T., Hucho F., Reutter W. (1991). The phospholipid analogue, hexadecylphosphocholine, inhibits protein kinase C in vitro and antagonises phorbol ester-stimulated cell proliferation.. Eur J Cancer.

[OCR_01144] Gescher A. (1985). Antiproliferative properties of phorbol ester tumour promoters.. Biochem Pharmacol.

[OCR_01148] Gschwendt M., Leibersperger H., Marks F. (1989). Differentiative action of K252a on protein kinase C and a calcium-unresponsive, phorbol ester/phospholipid-activated protein kinase.. Biochem Biophys Res Commun.

[OCR_01159] Hannun Y. A., Bell R. M. (1988). Aminoacridines, potent inhibitors of protein kinase C.. J Biol Chem.

[OCR_01162] Hannun Y. A., Bell R. M. (1989). Functions of sphingolipids and sphingolipid breakdown products in cellular regulation.. Science.

[OCR_01154] Hannun Y. A., Bell R. M. (1987). Lysosphingolipids inhibit protein kinase C: implications for the sphingolipidoses.. Science.

[OCR_01167] Hannun Y. A., Foglesong R. J., Bell R. M. (1989). The adriamycin-iron(III) complex is a potent inhibitor of protein kinase C.. J Biol Chem.

[OCR_01172] Hass R., Pfannkuche H. J., Kharbanda S., Gunji H., Meyer G., Hartmann A., Hidaka H., Resch K., Kufe D., Goppelt-Strübe M. (1991). Protein kinase C activation and protooncogene expression in differentiation/retrodifferentiation of human U-937 leukemia cells.. Cell Growth Differ.

[OCR_01178] Hecker E. (1985). Cell membrane associated protein kinase C as receptor of diterpene ester co-carcinogens of the tumor promoter type and the phenotypic expression of tumors.. Arzneimittelforschung.

[OCR_01183] Heesbeen E. C., Verdonck L. F., Hermans S. W., van Heugten H. G., Staal G. E., Rijksen G. (1991). Alkyllysophospholipid ET-18-OCH3 acts as an activator of protein kinase C in HL-60 cells.. FEBS Lett.

[OCR_01189] Hennings H., Blumberg P. M., Pettit G. R., Herald C. L., Shores R., Yuspa S. H. (1987). Bryostatin 1, an activator of protein kinase C, inhibits tumor promotion by phorbol esters in SENCAR mouse skin.. Carcinogenesis.

[OCR_01195] Herbert J. M., Augereau J. M., Gleye J., Maffrand J. P. (1990). Chelerythrine is a potent and specific inhibitor of protein kinase C.. Biochem Biophys Res Commun.

[OCR_01200] Hess A. D., Silanskis M. K., Esa A. H., Pettit G. R., May W. S. (1988). Activation of human T lymphocytes by bryostatin.. J Immunol.

[OCR_01205] Hidaka H., Inagaki M., Kawamoto S., Sasaki Y. (1984). Isoquinolinesulfonamides, novel and potent inhibitors of cyclic nucleotide dependent protein kinase and protein kinase C.. Biochemistry.

[OCR_01211] Hirai M., Gamou S., Kobayashi M., Shimizu N. (1989). Lung cancer cells often express high levels of protein kinase C activity.. Jpn J Cancer Res.

[OCR_01216] Hocevar B. A., Fields A. P. (1991). Selective translocation of beta II-protein kinase C to the nucleus of human promyelocytic (HL60) leukemia cells.. J Biol Chem.

[OCR_01221] Hooper W. C., Abraham R. T., Ashendel C. L., Woloschak G. E. (1989). Differential responsiveness to phorbol esters correlates with differential expression of protein kinase C in KG-1 and KG-1a human myeloid leukemia cells.. Biochim Biophys Acta.

[OCR_01227] Hornung R. L., Pearson J. W., Beckwith M., Longo D. L. (1992). Preclinical evaluation of bryostatin as an anticancer agent against several murine tumor cell lines: in vitro versus in vivo activity.. Cancer Res.

[OCR_01233] House C., Kemp B. E. (1987). Protein kinase C contains a pseudosubstrate prototope in its regulatory domain.. Science.

[OCR_01238] House C., Wettenhall R. E., Kemp B. E. (1987). The influence of basic residues on the substrate specificity of protein kinase C.. J Biol Chem.

[OCR_01243] Housey G. M., Johnson M. D., Hsiao W. L., O'Brian C. A., Murphy J. P., Kirschmeier P., Weinstein I. B. (1988). Overproduction of protein kinase C causes disordered growth control in rat fibroblasts.. Cell.

[OCR_01249] Huang F. L., Yoshida Y., Cunha-Melo J. R., Beaven M. A., Huang K. P. (1989). Differential down-regulation of protein kinase C isozymes.. J Biol Chem.

[OCR_01254] Huang K. P., Huang F. L. (1990). Differential sensitivity of protein kinase C isozymes to phospholipid-induced inactivation.. J Biol Chem.

[OCR_01259] Huwiler A., Fabbro D., Pfeilschifter J. (1991). Differential recovery of protein kinase C-alpha and -epsilon isozymes after long-term phorbol ester treatment in rat renal mesangial cells.. Biochem Biophys Res Commun.

[OCR_01265] Isakov N., McMahon P., Altman A. (1990). Selective post-transcriptional down-regulation of protein kinase C isoenzymes in leukemic T cells chronically treated with phorbol ester.. J Biol Chem.

[OCR_01271] Isonishi S., Andrews P. A., Howell S. B. (1990). Increased sensitivity to cis-diamminedichloroplatinum(II) in human ovarian carcinoma cells in response to treatment with 12-O-tetradecanoylphorbol 13-acetate.. J Biol Chem.

[OCR_01277] Kase H., Iwahashi K., Nakanishi S., Matsuda Y., Yamada K., Takahashi M., Murakata C., Sato A., Kaneko M. (1987). K-252 compounds, novel and potent inhibitors of protein kinase C and cyclic nucleotide-dependent protein kinases.. Biochem Biophys Res Commun.

[OCR_01284] Kennedy M. J., Prestigiacomo L. J., Tyler G., May W. S., Davidson N. E. (1992). Differential effects of bryostatin 1 and phorbol ester on human breast cancer cell lines.. Cancer Res.

[OCR_01461] Kikkawa U., Kishimoto A., Nishizuka Y. (1989). The protein kinase C family: heterogeneity and its implications.. Annu Rev Biochem.

[OCR_01290] Kiley S., Schaap D., Parker P., Hsieh L. L., Jaken S. (1990). Protein kinase C heterogeneity in GH4C1 rat pituitary cells. Characterization of a Ca2(+)-independent phorbol ester receptor.. J Biol Chem.

[OCR_01296] Kobayashi E., Nakano H., Morimoto M., Tamaoki T. (1989). Calphostin C (UCN-1028C), a novel microbial compound, is a highly potent and specific inhibitor of protein kinase C.. Biochem Biophys Res Commun.

[OCR_01302] Komada F., Nishikawa M., Uemura Y., Morita K., Hidaka H., Shirakawa S. (1991). Expression of three major protein kinase C isozymes in various types of human leukemic cells.. Cancer Res.

[OCR_01310] Kopp R., Noelke B., Sauter G., Schildberg F. W., Paumgartner G., Pfeiffer A. (1991). Altered protein kinase C activity in biopsies of human colonic adenomas and carcinomas.. Cancer Res.

[OCR_01320] Kraft A. S., Anderson W. B. (1983). Phorbol esters increase the amount of Ca2+, phospholipid-dependent protein kinase associated with plasma membrane.. Nature.

[OCR_01314] Kraft A. S., Reeves J. A., Ashendel C. L. (1988). Differing modulation of protein kinase C by bryostatin 1 and phorbol esters in JB6 mouse epidermal cells.. J Biol Chem.

[OCR_01331] Kraft A. S., Smith J. B., Berkow R. L. (1986). Bryostatin, an activator of the calcium phospholipid-dependent protein kinase, blocks phorbol ester-induced differentiation of human promyelocytic leukemia cells HL-60.. Proc Natl Acad Sci U S A.

[OCR_01325] Krauss R. S., Housey G. M., Johnson M. D., Weinstein I. B. (1989). Disturbances in growth control and gene expression in a C3H/10T1/2 cell line that stably overproduces protein kinase C.. Oncogene.

[OCR_01345] Laughton C. A., Bradshaw T. D., Gescher A. (1989). Sterically hindered analogues of diacylglycerols. Synthesis, binding to the phorbol ester receptor and metabolism in A549 human lung carcinoma cells.. Int J Cancer.

[OCR_01338] Laughton C. A., Dale I. L., Gescher A. (1989). Studies on bioactive compounds. 13. Synthesis and lack of growth-inhibitory properties of cyclohexane-1,2,4-triol 1,2-diesters, which resemble ring C of the phorbol ester molecule.. J Med Chem.

[OCR_01351] Lilly M., Brown C., Pettit G., Kraft A. (1991). Bryostatin 1: a potential anti-leukemic agent for chronic myelomonocytic leukemia.. Leukemia.

[OCR_01358] Lindner D., Gschwendt M., Marks F. (1991). Down-regulation of protein kinase C in Swiss 3T3 fibroblasts is independent of its phosphorylating activity.. Biochem Biophys Res Commun.

[OCR_01364] Loomis C. R., Bell R. M. (1988). Sangivamycin, a nucleoside analogue, is a potent inhibitor of protein kinase C.. J Biol Chem.

[OCR_01369] Mackanos E. A., Pettit G. R., Ramsdell J. S. (1991). Bryostatins selectively regulate protein kinase C-mediated effects on GH4 cell proliferation.. J Biol Chem.

[OCR_01374] Mahoney C. W., Azzi A., Huang K. P. (1990). Effects of suramin, an anti-human immunodeficiency virus reverse transcriptase agent, on protein kinase C. Differential activation and inhibition of protein kinase C isozymes.. J Biol Chem.

[OCR_01380] Marais R. M., Parker P. J. (1989). Purification and characterisation of bovine brain protein kinase C isotypes alpha, beta and gamma.. Eur J Biochem.

[OCR_01385] Marx M. H., Piantadosi C., Noseda A., Daniel L. W., Modest E. J. (1988). Synthesis and evaluation of neoplastic cell growth inhibition of 1-N-alkylamide analogues of glycero-3-phosphocholine.. J Med Chem.

[OCR_01393] May W. S., Sharkis S. J., Esa A. H., Gebbia V., Kraft A. S., Pettit G. R., Sensenbrenner L. L. (1987). Antineoplastic bryostatins are multipotential stimulators of human hematopoietic progenitor cells.. Proc Natl Acad Sci U S A.

[OCR_01397] McBain J. A., Pettit G. R., Mueller G. C. (1988). Bryostatin 1 antagonizes the terminal differentiating action of 12-O-tetradecanoylphorbol-13-acetate in a human colon cancer cell.. Carcinogenesis.

[OCR_01403] McSwine-Kennick R. L., McKeegan E. M., Johnson M. D., Morin M. J. (1991). Phorbol diester-induced alterations in the expression of protein kinase C isozymes and their mRNAs. Analysis in wild-type and phorbol diester-resistant HL-60 cell clones.. J Biol Chem.

[OCR_01409] Megidish T., Mazurek N. (1989). A mutant protein kinase C that can transform fibroblasts.. Nature.

[OCR_01413] Merrill A. H., Nimkar S., Menaldino D., Hannun Y. A., Loomis C., Bell R. M., Tyagi S. R., Lambeth J. D., Stevens V. L., Hunter R. (1989). Structural requirements for long-chain (sphingoid) base inhibition of protein kinase C in vitro and for the cellular effects of these compounds.. Biochemistry.

[OCR_01421] Meyer T., Regenass U., Fabbro D., Alteri E., Rösel J., Müller M., Caravatti G., Matter A. (1989). A derivative of staurosporine (CGP 41 251) shows selectivity for protein kinase C inhibition and in vitro anti-proliferative as well as in vivo anti-tumor activity.. Int J Cancer.

[OCR_01428] Mochly-Rosen D., Khaner H., Lopez J., Smith B. L. (1991). Intracellular receptors for activated protein kinase C. Identification of a binding site for the enzyme.. J Biol Chem.

[OCR_01433] Molleyres L. P., Rando R. R. (1988). Structural studies on the diglyceride-mediated activation of protein kinase C.. J Biol Chem.

[OCR_01438] Mori T., Takai Y., Minakuchi R., Yu B., Nishizuka Y. (1980). Inhibitory action of chlorpromazine, dibucaine, and other phospholipid-interacting drugs on calcium-activated, phospholipid-dependent protein kinase.. J Biol Chem.

[OCR_01444] Morin M. J., Kreutter D., Rasmussen H., Sartorelli A. C. (1987). Disparate effects of activators of protein kinase C on HL-60 promyelocytic leukemia cell differentiation.. J Biol Chem.

[OCR_01450] Nishikawa M., Komada F., Uemura Y., Hidaka H., Shirakawa S. (1990). Decreased expression of type II protein kinase C in HL-60 variant cells resistant to induction of cell differentiation by phorbol diester.. Cancer Res.

[OCR_01456] Nishizuka Y. (1988). The molecular heterogeneity of protein kinase C and its implications for cellular regulation.. Nature.

[OCR_01471] O'Brian C. A., Liskamp R. M., Solomon D. H., Weinstein I. B. (1985). Inhibition of protein kinase C by tamoxifen.. Cancer Res.

[OCR_01476] O'Brian C., Vogel V. G., Singletary S. E., Ward N. E. (1989). Elevated protein kinase C expression in human breast tumor biopsies relative to normal breast tissue.. Cancer Res.

[OCR_01465] Obeid L. M., Okazaki T., Karolak L. A., Hannun Y. A. (1990). Transcriptional regulation of protein kinase C by 1,25-dihydroxyvitamin D3 in HL-60 cells.. J Biol Chem.

[OCR_01482] Okoshi H., Hakomori S., Nisar M., Zhou Q. H., Kimura S., Tashiro K., Igarashi Y. (1991). Cell membrane signaling as target in cancer therapy. II: Inhibitory effect of N,N,N-trimethylsphingosine on metastatic potential of murine B16 melanoma cell line through blocking of tumor cell-dependent platelet aggregation.. Cancer Res.

[OCR_01490] Ono Y., Fujii T., Ogita K., Kikkawa U., Igarashi K., Nishizuka Y. (1989). Protein kinase C zeta subspecies from rat brain: its structure, expression, and properties.. Proc Natl Acad Sci U S A.

[OCR_01496] Parker P. J., Kour G., Marais R. M., Mitchell F., Pears C., Schaap D., Stabel S., Webster C. (1989). Protein kinase C--a family affair.. Mol Cell Endocrinol.

[OCR_01501] Pasti G., Rivedal E., Yuspa S. H., Herald C. L., Pettit G. R., Blumberg P. M. (1988). Contrasting duration of inhibition of cell-cell communication in primary mouse epidermal cells by phorbol 12,13-dibutyrate and by bryostatin 1.. Cancer Res.

[OCR_01508] Pears C., Parker P. J. (1991). Down-regulation of a kinase defective PKC-alpha.. FEBS Lett.

[OCR_01512] Pearson J. D., DeWald D. B., Mathews W. R., Mozier N. M., Zürcher-Neely H. A., Heinrikson R. L., Morris M. A., McCubbin W. D., McDonald J. R., Fraser E. D. (1990). Amino acid sequence and characterization of a protein inhibitor of protein kinase C.. J Biol Chem.

[OCR_01520] Pelosin J. M., Keramidas M., Souvignet C., Chambaz E. M. (1990). Differential inhibition of protein kinase C subtypes.. Biochem Biophys Res Commun.

[OCR_01525] Persons D. A., Wilkison W. O., Bell R. M., Finn O. J. (1988). Altered growth regulation and enhanced tumorigenicity of NIH 3T3 fibroblasts transfected with protein kinase C-I cDNA.. Cell.

[OCR_01537] Pettit G. R., Day J. F., Hartwell J. L., Wood H. B. (1970). Antineoplastic components of marine animals.. Nature.

[OCR_01547] Pontremoli S., Melloni E., Sparatore B., Michetti M., Salamino F., Horecker B. L. (1990). Isozymes of protein kinase C in human neutrophils and their modification by two endogenous proteinases.. J Biol Chem.

[OCR_01553] Posada J. A., McKeegan E. M., Worthington K. F., Morin M. J., Jaken S., Tritton T. R. (1989). Human multidrug resistant KB cells overexpress protein kinase C: involvement in drug resistance.. Cancer Commun.

[OCR_01559] Powis G. (1991). Signalling targets for anticancer drug development.. Trends Pharmacol Sci.

[OCR_01563] Rando R. R., Kishi Y. (1992). Structural basis of protein kinase C activation by diacylglycerols and tumor promoters.. Biochemistry.

[OCR_01568] Reddy A. L., Fialkow P. J. (1990). Evidence that weak promotion of carcinogen-initiated cells prevents their progression to malignancy.. Carcinogenesis.

[OCR_01573] Rodriguez-Pena A., Rozengurt E. (1984). Disappearance of Ca2+-sensitive, phospholipid-dependent protein kinase activity in phorbol ester-treated 3T3 cells.. Biochem Biophys Res Commun.

[OCR_01579] Rotenberg S. A., Smiley S., Ueffing M., Krauss R. S., Chen L. B., Weinstein I. B. (1990). Inhibition of rodent protein kinase C by the anticarcinoma agent dequalinium.. Cancer Res.

[OCR_01585] Ryves W. J., Evans A. T., Olivier A. R., Parker P. J., Evans F. J. (1991). Activation of the PKC-isotypes alpha, beta 1, gamma, delta and epsilon by phorbol esters of different biological activities.. FEBS Lett.

[OCR_01591] Sakanoue Y., Hatada T., Kusunoki M., Yanagi H., Yamamura T., Utsunomiya J. (1991). Protein kinase C activity as marker for colorectal cancer.. Int J Cancer.

[OCR_01597] Sako T., Yuspa S. H., Herald C. L., Pettit G. R., Blumberg P. M. (1987). Partial parallelism and partial blockade by bryostatin 1 of effects of phorbol ester tumor promoters on primary mouse epidermal cells.. Cancer Res.

[OCR_01610] Schaap D., Hsuan J., Totty N., Parker P. J. (1990). Proteolytic activation of protein kinase C-epsilon.. Eur J Biochem.

[OCR_01615] Schaap D., Parker P. J., Bristol A., Kriz R., Knopf J. (1989). Unique substrate specificity and regulatory properties of PKC-epsilon: a rationale for diversity.. FEBS Lett.

[OCR_01605] Schaap D., Parker P. J. (1990). Expression, purification, and characterization of protein kinase C-epsilon.. J Biol Chem.

[OCR_01621] Schuchter L. M., Esa A. H., May S., Laulis M. K., Pettit G. R., Hess A. D. (1991). Successful treatment of murine melanoma with bryostatin 1.. Cancer Res.

[OCR_01625] Schwartz G. K., Redwood S. M., Ohnuma T., Holland J. F., Droller M. J., Liu B. C. (1990). Inhibition of invasion of invasive human bladder carcinoma cells by protein kinase C inhibitor staurosporine.. J Natl Cancer Inst.

[OCR_01631] Sekiguchi K., Tsukuda M., Ogita K., Kikkawa U., Nishizuka Y. (1987). Three distinct forms of rat brain protein kinase C: differential response to unsaturated fatty acids.. Biochem Biophys Res Commun.

[OCR_01637] Shimizu N., Ohta M., Fujiwara C., Sagara J., Mochizuki N., Oda T., Utiyama H. (1991). Expression of a novel immediate early gene during 12-O-tetradecanoylphorbol-13-acetate-induced macrophagic differentiation of HL-60 cells.. J Biol Chem.

[OCR_01644] Shimizu Y., Shimizu N. (1989). Cell genetic evidence of correlation of intracellular translocation of protein kinase C (PKC) and PKC-mediated phosphorylation of 80-kDa protein with mitogenic action of tumor promoters.. Somat Cell Mol Genet.

[OCR_01651] Smith J. B., Smith L., Pettit G. R. (1985). Bryostatins: potent, new mitogens that mimic phorbol ester tumor promoters.. Biochem Biophys Res Commun.

[OCR_01656] Stabel S., Parker P. J. (1991). Protein kinase C.. Pharmacol Ther.

[OCR_01662] Stevens V. L., Nimkar S., Jamison W. C., Liotta D. C., Merrill A. H. (1990). Characteristics of the growth inhibition and cytotoxicity of long-chain (sphingoid) bases for Chinese hamster ovary cells: evidence for an involvement of protein kinase C.. Biochim Biophys Acta.

[OCR_01667] Strulovici B., Daniel-Issakani S., Baxter G., Knopf J., Sultzman L., Cherwinski H., Nestor J., Webb D. R., Ransom J. (1991). Distinct mechanisms of regulation of protein kinase C epsilon by hormones and phorbol diesters.. J Biol Chem.

[OCR_01674] Sullivan J. P., Connor J. R., Shearer B. G., Burch R. M. (1991). 2,6-Diamino-N-([1-oxotridecyl)-2-piperidinyl]methyl)hexanamide (NPC 15437): a selective inhibitor of protein kinase C.. Agents Actions.

[OCR_01680] Tamaoki T., Nakano H. (1990). Potent and specific inhibitors of protein kinase C of microbial origin.. Biotechnology (N Y).

[OCR_01685] Tamaoki T., Nomoto H., Takahashi I., Kato Y., Morimoto M., Tomita F. (1986). Staurosporine, a potent inhibitor of phospholipid/Ca++dependent protein kinase.. Biochem Biophys Res Commun.

[OCR_01691] Trilivas I., McDonough P. M., Brown J. H. (1991). Dissociation of protein kinase C redistribution from the phosphorylation of its substrates.. J Biol Chem.

[OCR_01696] Tritton T. R., Hickman J. A. (1990). How to kill cancer cells: membranes and cell signaling as targets in cancer chemotherapy.. Cancer Cells.

[OCR_01707] Uberall F., Maly K., Egle A., Doppler W., Hofmann J., Grunicke H. H. (1991). Inhibition of cell proliferation, protein kinase C, and phorbol ester-induced fos expression by the dihydropyridine derivative B859-35.. Cancer Res.

[OCR_01701] Uberall F., Oberhuber H., Maly K., Zaknun J., Demuth L., Grunicke H. H. (1991). Hexadecylphosphocholine inhibits inositol phosphate formation and protein kinase C activity.. Cancer Res.

[OCR_01712] Valette A., Gas N., Jozan S., Roubinet F., Dupont M. A., Bayard F. (1987). Influence of 12-O-tetradecanoylphorbol-13-acetate on proliferation and maturation of human breast carcinoma cells (MCF-7): relationship to cell cycle events.. Cancer Res.

[OCR_01719] Vandenbark G. R., Niedel J. E. (1984). Phorbol diesters and cellular differentiation.. J Natl Cancer Inst.

[OCR_01723] Warren B. S., Kamano Y., Pettit G. R., Blumberg P. M. (1988). Mimicry of bryostatin 1 induced phosphorylation patterns in HL-60 cells by high-phorbol ester concentrations.. Cancer Res.

[OCR_01729] Weinstein I. B. (1988). The origins of human cancer: molecular mechanisms of carcinogenesis and their implications for cancer prevention and treatment--twenty-seventh G.H.A. Clowes memorial award lecture.. Cancer Res.

[OCR_01735] Wender P. A., Cribbs C. M., Koehler K. F., Sharkey N. A., Herald C. L., Kamano Y., Pettit G. R., Blumberg P. M. (1988). Modeling of the bryostatins to the phorbol ester pharmacophore on protein kinase C.. Proc Natl Acad Sci U S A.

[OCR_01742] Wise B. C., Kuo J. F. (1983). Modes of inhibition by acylcarnitines, adriamycin and trifluoperazine of cardiac phospholipid-sensitive calcium-dependent protein kinase.. Biochem Pharmacol.

[OCR_01748] Wolfman A., Macara I. G. (1987). Elevated levels of diacylglycerol and decreased phorbol ester sensitivity in ras-transformed fibroblasts.. Nature.

[OCR_01753] Yamanishi D. T., Graham M., Buckmeier J. A., Meyskens F. L. (1991). The differential expression of protein kinase C genes in normal human neonatal melanocytes and metastatic melanomas.. Carcinogenesis.

[OCR_01764] Yoshizawa S., Fujiki H., Suguri H., Suganuma M., Nakayasu M., Matsushima R., Sugimura T. (1990). Tumor-promoting activity of staurosporine, a protein kinase inhibitor on mouse skin.. Cancer Res.

[OCR_01759] Young S., Parker P. J., Ullrich A., Stabel S. (1987). Down-regulation of protein kinase C is due to an increased rate of degradation.. Biochem J.

[OCR_01770] Zheng B., Oishi K., Shoji M., Eibl H., Berdel W. E., Hajdu J., Vogler W. R., Kuo J. F. (1990). Inhibition of protein kinase C, (sodium plus potassium)-activated adenosine triphosphatase, and sodium pump by synthetic phospholipid analogues.. Cancer Res.

[OCR_01091] de Vries D. J., Herald C. L., Pettit G. R., Blumberg P. M. (1988). Demonstration of sub-nanomolar affinity of bryostatin 1 for the phorbol ester receptor in rat brain.. Biochem Pharmacol.

